# An integrated bioinformatic investigation of mitochondrial solute carrier family 25 (SLC25) in colon cancer followed by preliminary validation of member 5 (SLC25A5) in tumorigenesis

**DOI:** 10.1038/s41419-022-04692-1

**Published:** 2022-03-14

**Authors:** Yan-Jie Chen, Wei-Feng Hong, Meng-Ling Liu, Xi Guo, Yi-Yi Yu, Yue-Hong Cui, Tian-Shu Liu, Li Liang

**Affiliations:** 1 Department of Gastroenterology, Zhongshan Hospital Affiliated to Fudan University, NO. 180, Fenglin Road, Xuhui District, Shanghai, 200032 People’s Republic of China; 2 Department of Radiation Oncology, Zhongshan Hospital Affiliated to Fudan University, Shanghai, 200032 People’s Republic of China; 3 Department of Medical Oncology, Zhongshan Hospital Affiliated to Fudan University, NO. 180, Fenglin Road, Xuhui District, Shanghai, 200032 People’s Republic of China; 4 Cancer Center, Zhongshan Hospital Affiliated to Fudan University, NO. 180, Fenglin Road, Xuhui District, Shanghai, 200032 People’s Republic of China; 5 Evidence-Based Medicine Center, Zhongshan Hospital Affiliated to Fudan University, NO. 180, Fenglin Road, Xuhui District, Shanghai, 200032 People’s Republic of China

**Keywords:** Metabolomics, Cancer metabolism, Oncogenes, Colon cancer, Tumour biomarkers

## Abstract

Solute carrier family 25 (SLC25) encodes transport proteins at the inner mitochondrial membrane and functions as carriers for metabolites. Although SLC25 genetic variants correlate with human metabolic diseases, their roles in colon cancer remain unknown. Cases of colon cancer were retrieved from The Cancer Genome Atlas, and the transcriptionally differentially expressed members (DEMs) of SLC25 were identified. DNA level alterations, clinicopathological characteristics, and clinical survival were also investigated. A risk score model based on the DEMs was constructed to further evaluate their prognostic values in a clinical setting. The results were preliminarily validated using bioinformatic analysis of datasets from the Gene Expression Omnibus, immunohistochemical evaluations in clinical specimens, and functional experiments in colon cancer-derived cell lines. Thirty-seven DEMs were identified among 53 members of SLC25. Eight of 37 DEMs were introduced into a risk score model using integrated LASSO regression and multivariate Cox regression. Validated by GSE395282 and GSE175356, DEMs with high-risk scores were associated with the phenotypes of increasing tumor immune infiltration and decreasing glycolysis and apoptosis contents. SLC25A5 was downregulated in cancer, and its upregulation was related to better overall survival in patients from public datasets and in clinical cases. High SLC25A5 expression was an independent prognostic factor for 79 patients after surgical treatment. A negative correlation between CD8 and SLC25A5 was determined in specimens from 106 patients with advanced colon cancer. SLC25A5 attenuated cell proliferation, upregulated the expression of programmed cell death-related signatures, and exerted its biological function by inhibiting the MAPK signaling pathway. Our study reveals that mitochondrial SLC25 has prognostic value in patients with colon cancer. The bioinformatic analyses by following verification in situ and in vitro provide direction for further functional and mechanistic studies on the identified member of SLC25.

## Introduction

Colorectal cancer (CRC) is common in the West and East and has relatively high morbidity and mortality [1, 2]. Since energy metabolism reprogramming, characterized by aerobic glycolysis and mitochondrial oxidative phosphorylation dysfunction, is a hallmark of cancer, understanding the novel molecular mechanism regulating mitochondrial metabolism in tumorigenesis, tumor growth, metastasis, and drug tolerance is critical for antitumor therapy development [3].

**Fig. 1:**
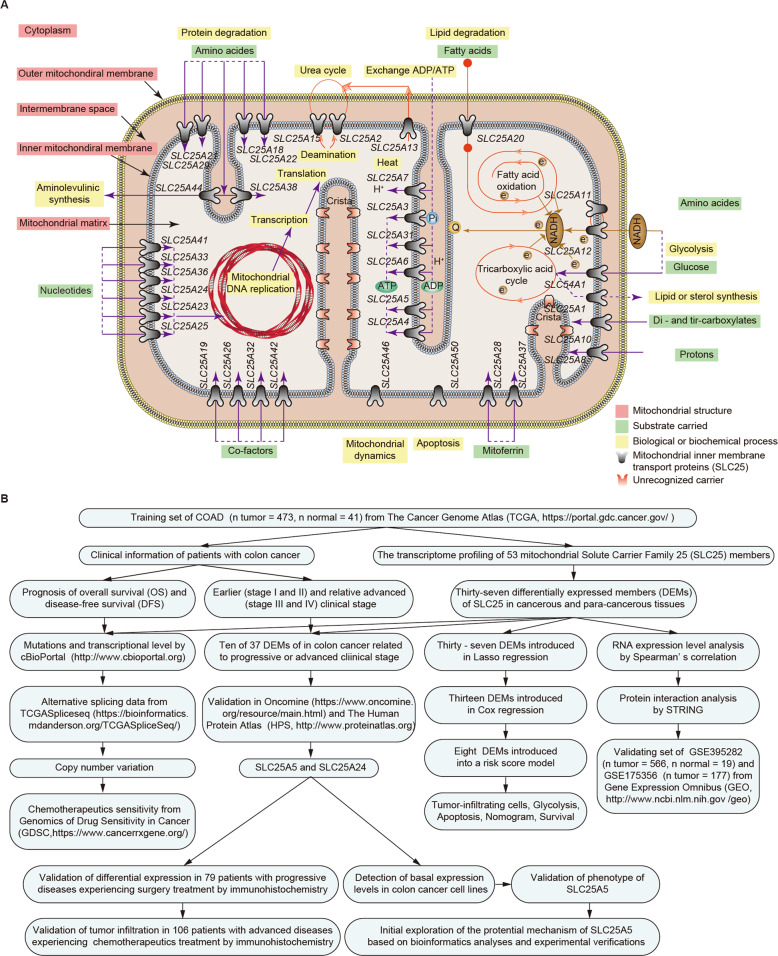
Solute carrier family 25 (SLC25) in mitochondrial metabolism and the flow chart of the study on its role in human colon cancer. **A** A simple diagram of metabolic substrates carried by mitochondrial SLC25 and related biochemical processes in mitochondria. **B** The scheme of this study.

Mitochondrial membrane transport proteins are located in the inner mitochondrial membrane and act as carriers for metabolic substrates (Fig. 1A). There are 53 transport proteins encoded by solute carrier family 25 (SLC25) in human beings (Supplementary Table S1) [4]. DNA mutations or aberrant expression of SLC25 causes cancerous diseases and noncancerous disorders via abnormal metabolism [5]. Horimoto reported that SLC25A8 is upregulated in colon cancer and is related to tumor differentiation [6]. Kuai found that SLC25A14 represses the increase in H_2_O_2_ products resulting from mitochondrial dysfunction in a feedback mechanism in colon cancer [7]. We previously demonstrated that SLC25A18 is a prognostic biomarker for patients with colon cancer and can suppress cell glycolysis and proliferation by suppressing Wnt/β-catenin cascades [8].

Hereby, we used datasets from public databases to comprehensively describe the genetic variations and transcriptomic alterations in SLC25. Then, we analyzed the differentially expressed members (DEMs) of SLC25 along with their function and tumor immune infiltration and constructed a prognostic risk model. Furthermore, we initially validated our findings in clinical specimens and explored the function of the identified member (SLC25A5) in colon cancer-derived cell lines.

## Results

### DEMs of mitochondrial SLC25 in colon cancer

**Fig. 2:**
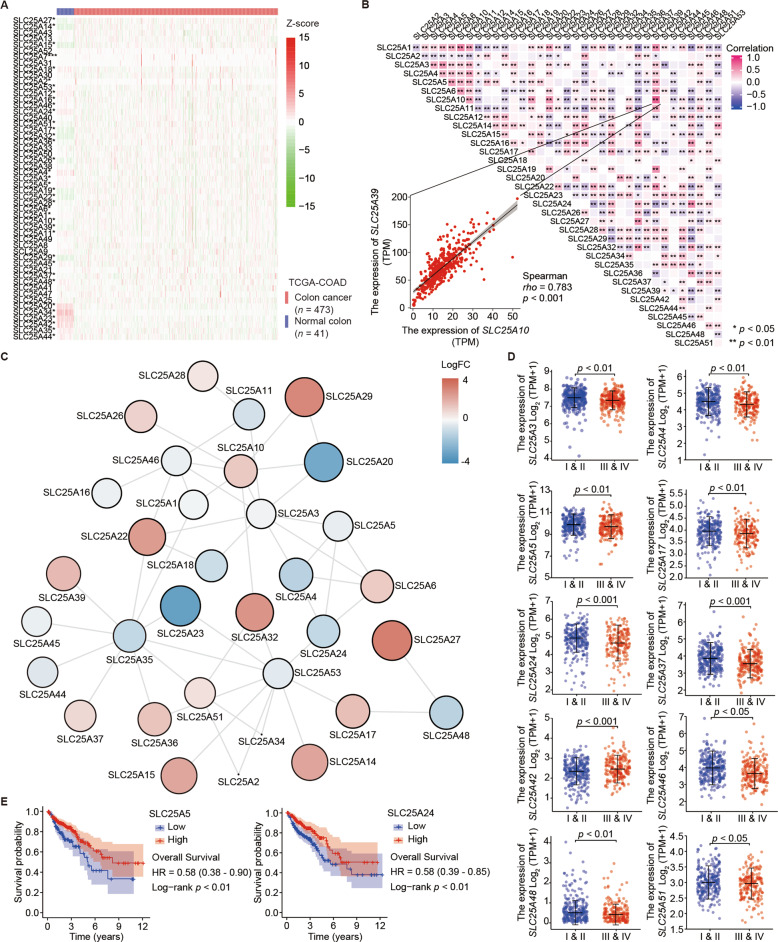
Expression of mitochondrial SLC25 in colon cancer. **A** Heatmap showing the expression of 53 SLC25 members between colon cancer and normal colon tissues according to the TCGA-COAD dataset. Thirty-seven of 53 were differentially expressed members (DEMs). **B** Spearman’s rank correlation analyses between 37 DEMs at the transcriptional level. Negative and positive correlations are marked blue and red, respectively. The scatter plot shows the correlation between SLC25A10 and SLC25A39 expression as an illustrative example. **C** Most of the 37 DEMs interacted at the protein level. The color of the nodes represents the differential expression level, while the size of the nodes indicates the degree of importance in the network. **D** Ten of 37 DEMs and stage I or II diseases were relevant in this analysis by Wilcoxon rank-sum test, showing that patients with earlier tumor stage tended to have higher mRNA expression of SLC25. **E** Kaplan–Meier curves suggested that patients with upregulated SLC25A5 and SLC24A24 had a longer overall survival (OS) time.

A detailed flow chart of this study is provided in Fig. 1B. First, we retrieved the row data of transcriptome profiling from the Genomic Data Commons (GDC) of the TCGA data portal, including 473 colon cancer samples and 41 normal colon samples. The baseline data of the training set are shown in Table S2. While GSE17636 contains 177 colon cancer samples, GSE39582 includes 566 colon cancer and 19 normal colon samples as the validation datasets. Both are based on the GPL570 (Affymetrix Human Genome U133 Plus 2.0 Array) platform. Thirty-seven DEMs were identified among all members of SLC25 in colon cancer (Fig. 2A). Of these, 18 DEMs were upregulated and 19 DEMs were downregulated in colon cancers compared to their levels in paracancerous colon tissues (Table S3). Positive (red) and negative (blue) correlations between two of 37 DEMs at the transcriptional level were inspected by Spearman’s rank correlation test (Fig. 2B), and the interactive relationships among the DEMs at the protein level were illustrated using a protein–protein interaction (PPI) network (Fig. 2C). To identify the correlations between DEMs and clinical parameters, we investigated the association between the expression levels of the 37 DEMs and clinical staging and prognosis. We found that the expression levels of 10 DEMs, including SLC25A3 (*p* < 0.01), SLC25A4 (*p* < 0.01), SLC25A5 (*p* < 0.01), SLC25A17 (*p* < 0.01), SLC25A24 (*p* < 0.001), SLC25A37 (*p* < 0.001), SLC25A42 (*p* < 0.001), SLC25A46 (*p* < 0.05), SLC25A48 (*p* < 0.01), and SLC25A51 (*p* < 0.05), were related to the clinical stage (Fig. 2D). Typical immunohistochemical staining images obtained from The Human Protein Atlas of 9 DEMs (lacking SLC25A3) in colon cancer specimens and normal colon tissues are shown (Fig. S1A). Their RNA expression levels (lacking SLC25A48) across cancers were surveyed in ONCOMINE (Fig. S1B). Although high expression of most DEMs indicated a longer survival time of patients with colon cancer (Fig. S1C), only that of SLC25A5 (HR = 0.58, log-rank *p* < 0.01, 95% CI 0.38–0.90) and SLC25A24 (HR = 0.58, log-rank *p* < 0.01, 95% CI 0.39–0.85) met statistical significance (Fig. 2E).

### Genetic variations and transcriptomic alterations in DEMs of mitochondrial SLC25 and the clinical prognostic and predictive value for patients with colon cancer

**Fig. 3:**
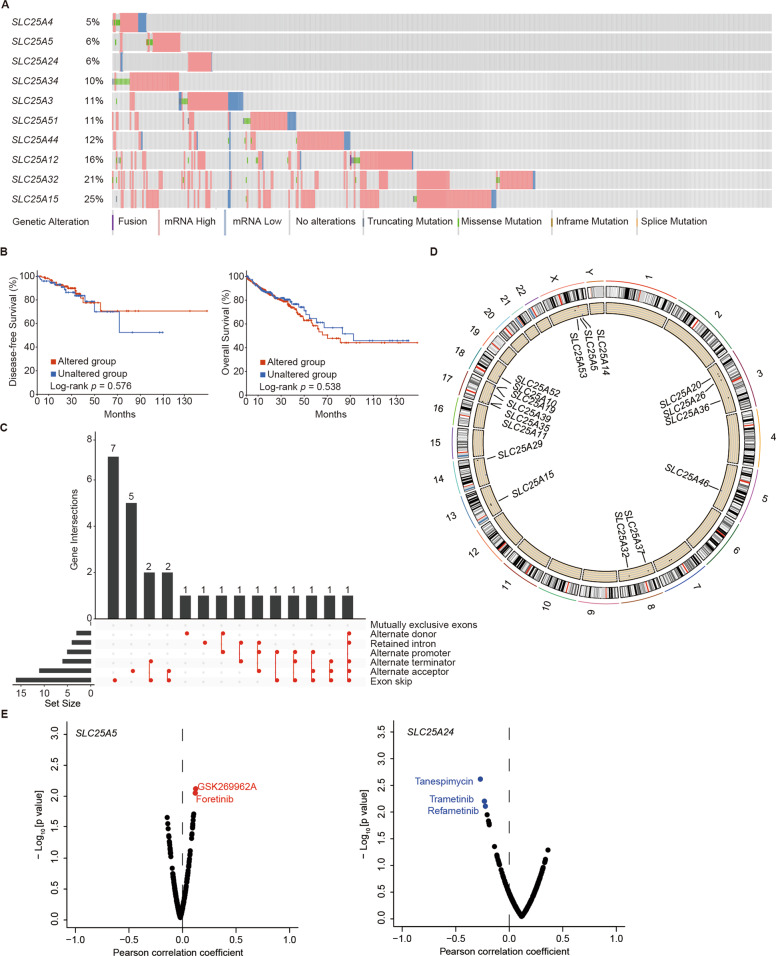
The genomic and transcriptomic variations of DEMs in colon cancer. **A** DEMs of SLC25 with differential DNA mutation rates and mRNA expression levels in colon cancer from the TCGA dataset were identified using cBioPortal. Among them, *SLC25A4, SLC25A5*, *SLC25A24*, *SLC25A34*, *SLC25A3*, *SLC25A51*, *SLC25A44*, *SLC25A12*, *SLC25A32*, and *SLC25A15* exhibited higher mutation frequencies, with rates of 5, 6, 6, 10, 11, 11, 12, 16, 21, and 25%, respectively. **B** No statistically significant differences in OS and disease-free survival (DFS) were observed in patients with colon cancer grouped by DNA mutation rate in the altered and unaltered groups by log-rank test, despite a longer overall survival time in the unaltered group with a lower DNA mutation frequency in DMEs of SLC25. **C** An UpSet diagram exhibited alternative splicing for DMEs and suggested that exon skipping was the major type of alternative splicing event. **D** The locations of copy number variation in DMEs of 23 SLC25 human chromosome pairs. **E** Patients with SLC25A5 overexpression showed a significantly high sensitivity to the ROCK inhibitor GSK269962A and to foretinib. Upregulation of SLC25A24 increased tansapimycin, trametinib, and refametinib resistance.

To determine the type of genetic variation in DEMs of SLC25, such as DNA mutation, RNA alternative splicing and CNV, we utilized cBioProtal and found that mutations in SLC25A4, SLC25A5, SLC25A24, SLC25A34, SLC25A3, SLC25A51, SLC25A44, SLC25A12, SLC25A32, and SLC25A15 occurred at a higher frequency in patients with colon cancer than in healthy individuals, and the rates were 5, 6, 6, 10, 11, 11, 12, 16, 21, and 25, respectively (Fig. 3A). Kaplan–Meier curves showed that patients with genetic alterations in DEMs had a longer overall survival time, regardless of the fact that no difference in disease-free survival (DFS) (*p* = 0.576) and overall survival (OS) (*p* = 0.538) was demonstrated using the log-rank test (Fig. 3B). Exon skipping was the dominant type of alternative splicing events (ASEs) in DEMs of SLC25 (Fig. 3C). The location of CNV alterations in DEMs on 23 human chromosome pairs is shown in Fig. 3D. To explore the role of SLC25 in antineoplastic drug sensitivity, we utilized GDSC and found that SLC25A5 and SLC25A24, which are highly expressed in colon cancer and indicative of a relatively longer survival time, were associated with the ROCK inhibitor GSK269962A and foretinib sensitivity and tanespimycin, trametinib, and refametinib resistance (Fig. 3E), respectively.

### Reclustering colon cancer and establishing a risk model based on RNA expression of mitochondrial SLC25

**Fig. 4:**
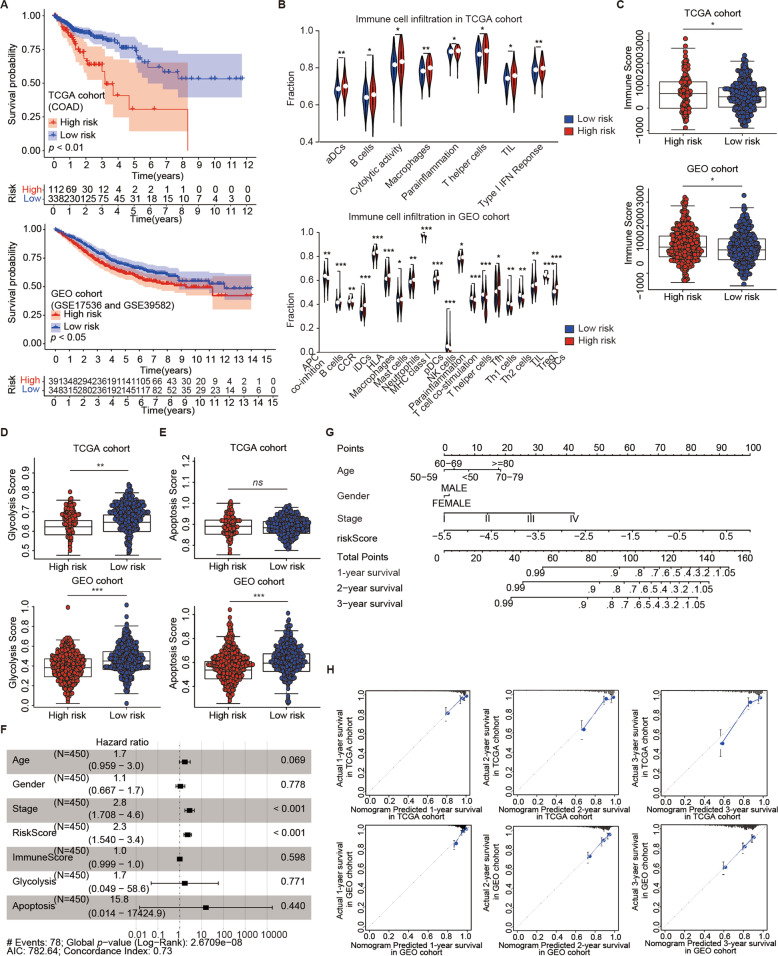
SLC25 members with high-risk scores are related to the phenotypes of increasing tumor immune infiltration and decreasing glycolysis and apoptosis in colon cancer and are involved in a nomogram for survival prediction. **A** Kaplan–Meier analysis showed that both in the training set from TCGA and validating sets from GSE17536 and GSE39582, patients with high-risk scores had a relatively short OS time. **B** Clinical cases with high-risk scores showed tumor immune infiltration. **C** Patients in the high-risk group had significantly higher immune scores than those in the low-risk group. Patients in the high-risk score group had significantly lower glycolysis (**D**) and apoptosis (**E**) pathway scores. **F** Risk score represented as an independent risk factor for predicting the prognosis for patients with colon cancer using multivariate Cox regression anand multivariate Cox regression analyses for overall sualysis. **G** A nomogram including risk scores showed estimates of the prognosis for cases from the TCGA database. **H** The calibration curve of the nomogram suggested an optimal agreement between the estimated overall survival outcomes at 1 year, 2 years, and 3 years and the actual observed clinical outcomes of the cases from TCGA and GEO datasets. Statistical analysis: Wilcoxon rank-sum test. *ns*, nonsignificant, *p < 0.05, **p < 0.01, ***p < 0.001, ****p < 0.0001.

To comprehensively evaluate the prognostic value of SLC25 in patients with colon cancer, we identified the optimal number and stability among all categories and re-clustered all cases into three subtypes (Fig. S2A). The new subtypes based on SLC25 expression and their relationship with clinicopathological characteristics are shown in Fig. S2B. This new categorization was unable to distinguish patients based on gene expression at the transcriptional level (Fig. S2C), regardless of the relatively long OS time observed in cluster C of SLC25 (*p* = 0.429, Fig. S2D). To further quantify the impact of SLC25 on the prognosis of all cases, we tried to construct a novel risk score model. Following LASSO regression analysis, 13 of 37 DEMs were identified as the most valuable prognostic predictors (Fig. S2E, F), eight of which were subsequently included in a Cox proportional hazard model with respective coeffective values (Fig. S2G). Then, the risk score for each case from TCGA was calculated by the eight DEMs. Kaplan–Meier curves suggested that patients with a high-risk score experienced a relatively short OS time based on cases from datasets of TCGA-COAD, GSE17536, and GSE39582 (Fig. 4A). The time-dependent receiver operating characteristic curve (ROC) revealed that the model presented powerful efficacy in predicting the OS of cases from TCGA, as the area under the curve (AUC) of 1-year, 3-year, and 5-year OS were 0.626, 0.676, and 0.749, respectively (Fig. S2H). The risk score distribution and gene expression pattern are shown in Fig. S2I. The time-dependent ROC analysis showed the predictive value of this model in the validation sets (Fig. S2J). The risk score distribution and gene expression pattern for the validation sets are shown in Fig. S2K.

### A high-risk score was related to the phenotype of boosting immune infiltration, low levels of tumor glycolysis and apoptosis, and prognostic value for patients

To understand the function of SLC25 in colon cancer, we focused on tumor immune infiltration, glucose metabolism, and apoptosis and found that the high-risk group was characterized by increasing tumor immune infiltration, as indicated in Fig. 4B. Patients in the high-risk group had significantly higher immune scores than those in the low-risk group (Fig. 4C). We further compared the glycolysis and apoptosis pathway scores between the two risk groups and found that the activity of glycolysis (Fig. 4D) and of the apoptosis pathway (Fig. 4E) were repressed in patients with high-risk scores. Using univariate and multivariate analyses (Table 1), the risk score (hazard ratio [HR] = 2.30, 95% confidence interval [CI] 1.54–3.43, *p* < 0.001) acted as an independent risk factor for predicting colon cancer prognosis (Fig. 4F). A nomogram was constructed including clinicopathological characteristics, such as age, sex, and staging, along with the risk score to estimate the prognosis for cases from TCGA (Fig. 4G). The nomogram exhibited a favorable degree of discrimination performance both in the TCGA (0.771, 95% CI 0.834–0.708) cohort and in the GEO (0.682, 95% CI 0.761–0.648) cohort by calculating the C-index. The calibration curve of the nomogram suggested an optimal agreement between the estimated OS outcomes at 1-year, 2-year, and 3-year and the actual observed clinical outcomes of the patients with colon cancer from both the TCGA and GEO databases (Fig. 4H). A nomogram based on the DEMs of SLC25 in the risk model was also constructed (Fig. S2L) and evaluated by the calibration curves based on cases from TCGA (Fig. S2M) and GEO (Fig. S2N).

**Table 1:** Univariate and multivariate Cox regression analyses for overall survival of patients with colon cancer from TCGA database based on risk score constructed by SLC25.

Variables	Univariate Cox analysis		Multivariate Cox analysis	
	HR (95% CI)	*p* value	HR (95% CI)	*p* value
Age (≥ 60 vs. < 60)	1.23 (0.74–2.07)	0.42	1.70 (0.96–3.02)	0.069
Gender (male vs. female)	1.22 (0.79–1.90)	0.36	1.07 (0.67–1.72)	0.778
Stage (III plus IV vs. I plus II)	3.02 (1.89–4.82)	0.01	2.81 (1.71–4.63)	<0.001
Risk score (high vs. low)	2.72 (1.88–3.94)	0.01	2.30 (1.54–3.43)	<0.001
Immune score (high vs. low)	1.00 (0.99–1.00)	0.86	1.00 (1.00–1.00)	0.598
Glycolysis score (high vs. low)	0.61 (0.06–6.47)	0.68	1.69 (0.05–58.61)	0.771
Apoptosis score (high vs. low)	5.06 (0.10–258.53)	0.42	15.80 (0.01–17424.89)	0.440

### In-depth bioinformatic analyses of SLC25A5 and SLC25A24 in colon cancer

**Fig. 5:**
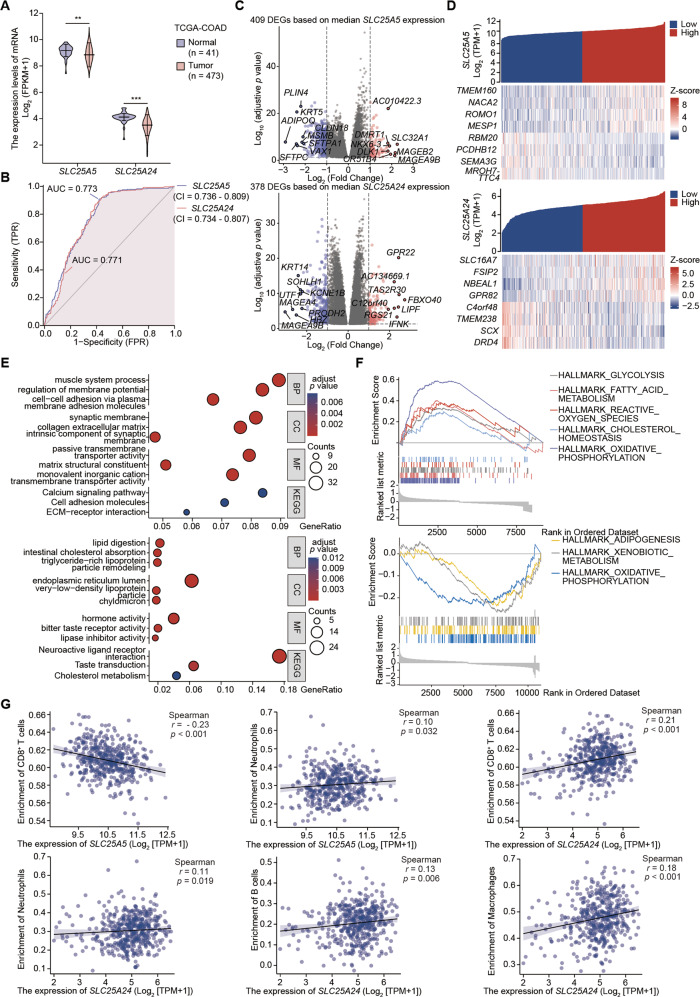
A further bioinformatic survey of SLC25A5 and SLC25A24 in colon cancer. **A** SLC25A5 and SLC25A24 were downregulated in colon cancer based on samples from TCGA-COAD. **B** Both exhibited favorable diagnostic biomarkers of colon cancer with an area under the curve over 0.7 in receiver operator characteristic curves. **C** Volcano plots showing differentially expressed genes (DEGs) between high- and low-expression stratifications of SLC25A5 and SLC25A24. DEGs with upregulated expressions are marked in red, and downregulated expressions are marked in blue. **D** Heatmaps revealed coexpressed genes based on the median expression levels of SLC25A5 and SLC25A24. The top eight coexpressed genes are displayed. **E** Bubble plots illustrating the terms of Gene Ontology (GO) and pathways in Kyoto Encyclopedia of Genes and Genomes (KEGG) enriched using DEGs identified by the expression of SLC25A5 (upper) and SLC25A24 (lower). **F** Gene sets involved in mitochondrial metabolism were enriched by differential expression levels of SLC25A5 (upper) and SLC25A24 (lower). **G** The expression of SLC25A5 was negatively related to CD8^+^ T cells (*r* = −0.23, *p* < 0.001) and positively related to neutrophils (*r* = 0.10, *p* < 0.05), while the expression of SLC25A24 was positively related to the infiltration of CD8^+^ T cells (*r* = 0.21, *p* < 0.001), neutrophils (*r* = 0.11, *p* < 0.05), B cells (*r* = 0.13, *p* < 0.01), and macrophages (*r* = 0.18, *p* < 0.001) by Spearman relation analysis. Statistical analysis: Wilcoxon rank-sum test, **p < 0.01, ***p < 0.001.

To further evaluate the clinical value of SLC25A5 and SLC25A24 in colon cancer, we performed a series of bioinformatic investigations. Although SLC25A5 and SLC25A24 displayed elevated expression compared to samples from The Genotype-Tissue Expression (GTEx) (Fig. S3A), both were relatively lowly expressed in colon cancer based on samples from TCGA-COAD (Fig. 5A), which requires further experimental validation. Regarding the diagnostic efficacy of colon cancer, the AUCs of ROCs were 0.773 (CI: 0.736–0.809) for SLC25A5 and 0.771 for SLC25A24 (CI: 0.734–0.807) (Fig. 5B). Then, we regrouped all the cases into high- and low-expression subtypes based on the median expression values of SLC25A5 and SLC25A24. We identified 409 and 378 DEGs, of which 91 were upregulated and 318 were downregulated for SLC25A5, while 139 were upregulated and 239 were downregulated for SLC25A24. The top sixteen DEGs with upregulated DEGs in red and downregulated DEGs in blue are shown in volcano plots in Fig. 5C. Meanwhile, we identified 1152 and 4403 coexpressed genes, of which 649 were positively related and 459 were negatively related to SLC25A5, while 3867 were positively related and 136 were negatively related to SLC25A24. The top eight coexpressed genes are shown in the heatmaps in Fig. 5D. Under threshold conditions, we identified 177 items for terms of biological process (BP), 69 items for terms of cellular component (CC), 38 items for terms of molecular function (MF), and 8 items for KEGG pathways based on the DEGs determined by the expression of SLC25A5, while we identified 131 items for terms of BP, 24 items for terms of CC, 28 items for terms of MF, and 4 items for KEGG pathways based on the DEGs determined by the expression of SLC25A24. The top three GO terms and KEGG pathways enriched using DEGs identified by the expression of SLC25A5 (upper) and SLC25A24 (lower) are illustrated by the bubble plots in Fig. 5E. The top five terms are listed in Supplementary Table S4, and the genes enriched in the terms are labeled in cycle diagrams (Fig. S3B). Gene sets involved in metabolism, such as HALLMARK_GLYCOLYSIS for SLC25A5 and HALLMARK_ADIPOGENESIS for SLC25A24, were enriched by differential expression levels of SLC25A5 (Fig. 5F, upper) and SLC25A24 (Fig. 5F, lower). In addition, gene sets enriched in cancer-related phenotypes and signaling pathways for SLC25A5 and SLC25A24 (Fig. S3C) were also investigated. After comprehensively inspecting the association between tumor immune infiltration of 23 common immune cells and the expression of SLC25A5 and SLC25A24 (Fig. S3D), we revealed that the expression of SLC25A5 was negatively related to the infiltration of CD8^+^ T cells (*r* = −0.23, *p* < 0.001) and positively related to neutrophils (*r* = 0.10, *p* < 0.05), while the expression of SLC25A24 was positively related to the infiltration of CD8^+^ T cells (*r* = 0.21, *p* < 0.001), neutrophils (*r* = 0.11, *p* < 0.05), B cells (*r* = 0.13, *p* < 0.01), and macrophages (*r* = 0.18, *p* < 0.001) (Fig. 5G). Lollipop plots suggested a correlation between their expression and hallmark GSEA sets (Fig. S4A, B).

### Validation of the intracellular localization, expression, prognostic value, and tumor immune infiltration of SLC25A5 and SLC25A24 in clinical specimens

**Fig. 6:**
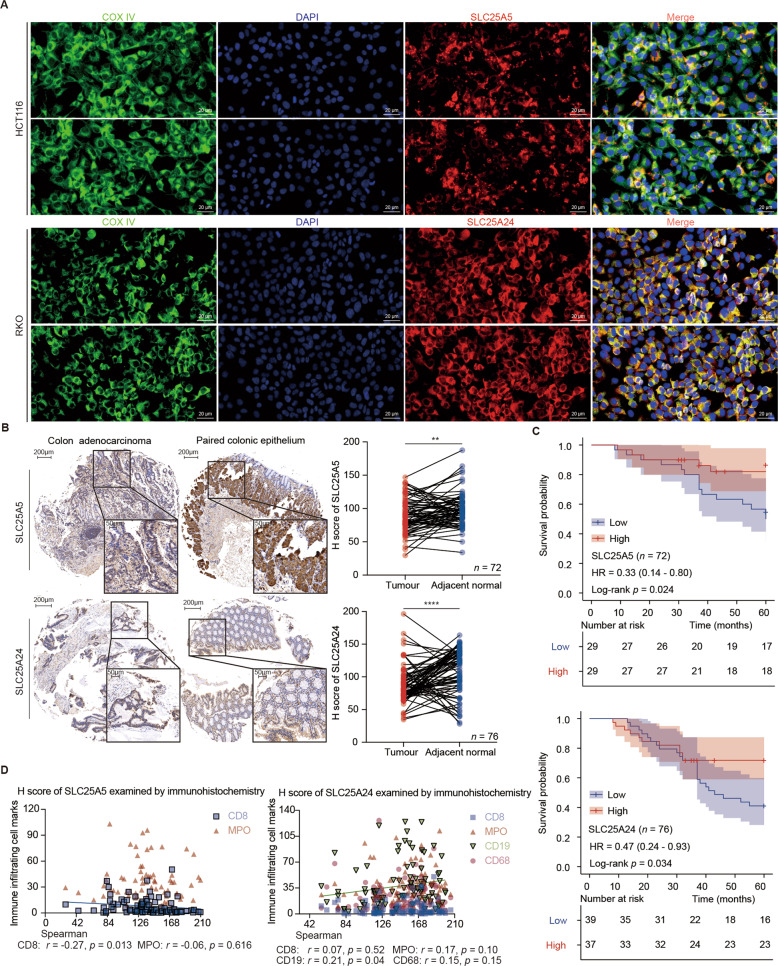
Validating expression of SLC25A5 and SLC25A24 in clinical specimens of colon cancer. **A** Immunofluorescence was used to determine the location of SLC25A5 and SLC25A24 in colon cancer-derived HCT116 and RKO cells. **B** Immunohistochemical examinations suggested that the expression levels of SLC25A5 (*n* = 72) and SLC25A24 (*n* = 76) were lower in colon cancer tissues than in adjacent normal tissues in patients receiving radical surgery resection. **C** Kaplan–Meier survival curves suggested that patients with upregulated SLC25A5 (HR = 0.33, *p* < 0.05) and SLC25A24 (HR = 0.47, *p* < 0.05) experienced a longer survival time after surgical treatment. **D** Spearman correlation analyses suggested that the expression of SLC25A5 negatively correlated with that of CD8 (Spearman *r* = −0.27, *p* < 0.05), while the expression of SLC25A24 positively correlated with that of CD19 (Spearman *r* = 0.21, *p* < 0.05). Scale bars are 20 μm in (**A**) and 50 μm or 200 μm in (**B**). Statistical analysis: Student’s T test, **p < 0.01, ***p < 0.001.

The intracellular localization of SLC25A5 and SLC25A24 was visualized by immunofluorescence colocalization (Fig. 6A). Among surgical resection specimens of colon adenocarcinoma and paired colonic epithelium, 72 and 78 of 79 cases with progressive disease were successfully stained and evaluated for SLC25A5 and SLC25A24, while 87 and 103 of 106 cases with advanced disease finished the same work. The baseline clinicopathological characteristics of patients with colon cancer are listed in Table S5 and Table S6. Representative pictures showed that SLC25A5 and SLC25A24 were differentially expressed in tumor and adjacent normal tissues (Fig. S5A). Low expression of SLC25A5 (*p* < 0.01) and SLC25A24 (*p* < 0.001) was observed in colon cancer compared to that in paired colonic epithelium (Fig. 6B). Kaplan–Meier curves evaluated the cumulative survival rate based on expression and suggested that patients with high expression of SLC25A5 (*p* = 0.024) and SLC25A24 (*p* = 0.034) experienced relatively longer OS (Fig. 6C). Univariate and multivariate Cox regression analyses integrating clinical characteristics and SLC25A5 and SLC25A24 suggested that SLC25A5 (HR = 0.44, *p* < 0.01, 95% CI 0.21–0.96) was an independent prognostic factor, while SLC25A24 (HR = 0.83, *p* = 0.63, 95% CI 0.39–1.77) did not meet the statistical significance (Table 2). We performed immunohistochemical staining for markers of infiltrated surrounding immune cells, including CD8^+^ T cells, neutrophils, B cells, and macrophages (Fig. S5B and Table S6), in serial sections. Spearman correlation analyses suggested that the expression of SLC25A5 negatively correlated with that of CD8 (Spearman *r* = −0.27, *p* < 0.05), while the expression of SLC25A24 positively correlated with that of CD19 (Spearman *r* = 0.21, *p* < 0.05) (Fig. 6D).

**Table 2:** Univariate and multivariate Cox regression analyses of clinicopathological features and SLC25A5 and SCL25A24 expression.

Variables	Univariate Cox analysis			Multivariate Cox analysis		
	*p* value	Hazard ratio	95% confidence interval	*p* value	Hazard ratio	95% confidence interval
Gender	0.085	1.739	0.926–3.263	/	/	/
Age	0.868	1.002	0.975–1.030	/	/	/
Tumor site	0.230	0.680	0.362–1.277	/	/	/
Pathological type	0.518	1.246	0.640–2.427	/	/	/
Pathological grade	0.026	2.057	1.091–3.880	0.098	1.862	0.891–3.891
Tumor size	0.148	1.590	0.848–2.983	/	/	/
Gross type	0.560	0.885	0.586–1.336	/	/	/
T stage	0.001	3.121	1.629–5.981	0.146	1.698	0.832–3.465
N stage	0.001	4.348	2.718–6.957	0.034	2.878	1.080–7.666
Num of lymph nodes	0.001	1.268	1.155–1.392	0.868	1.021	0.801–1.300
Preoperative CEA level	0.012	1.001	1.000–1.002	0.652	1.000	0.999–1.001
Preoperative CA199 level	0.001	1.006	1.002–1.009	0.934	1.000	0.993–1.007
Preoperative CA125 level	0.013	1.023	1.005–1.041	0.803	0.996	0.965–1.028
Preoperative AFP level	0.555	1.071	0.853–1.345	/	/	/
SLC25A5	0.001	0.275	0.141–0.535	0.040	0.444	0.205–0.963
SLC25A24	0.046	0.490	0.244–0.986	0.634	0.832	0.390–1.773

### Validation of functional phenotypes of SLC25A5 in colon cancer-derived cell lines

**Fig. 7:**
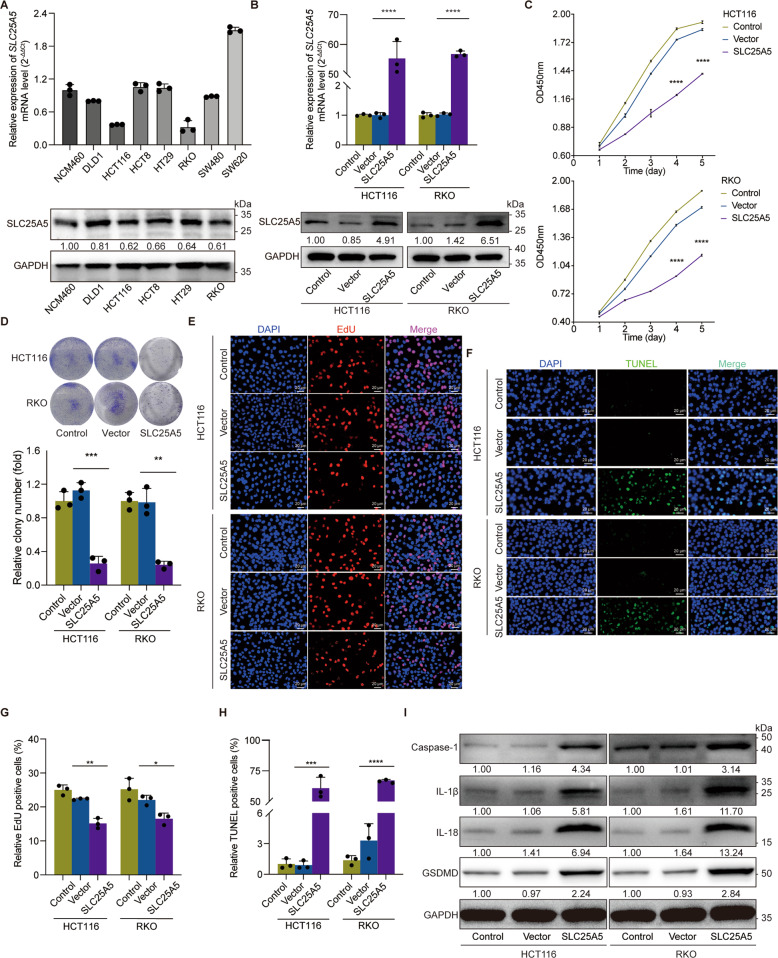
SLC25A5 repressed tumorigenesis and upregulated programmed cell death signaling in vitro. **A** The baseline expression of SLC25A5 at the mRNA and protein levels was examined in one human colonic epithelial cell line (NCM406) along with colon cancer cell lines (DLD1, HCT116, HCT8, HT29, RKO, SW480, SW620). **B** SLC25A5 was overexpressed by lentiviral transfection in HCT116 and RKO cells. Their expression levels were re-examined by RT–qPCR and western blotting. **C**, **D** Upregulation of SLC25A5 contributed to attenuating tumorigenesis, which was evaluated by CCK-8 assay (**C**), colony formation assay (**D**), and EdU (**E**) and TUNEL (**F**) staining. **G**, **H** Quantification of EdU and TUNEL staining. **I** Upregulation of SLC25A5 inhibited the expression of programmed cell death-related biomarkers, such as Caspase-1, IL-1β, IL-18, and GSDMD. GAPDH protein was used as the internal control. *n* = 3 biological replicates. Scale bar = 20 μm. Statistical analysis: Student’s T test, *p < 0.05, **p < 0.01, ***p < 0.001, ****p < 0.0001.

To explore the function of SLC25A5 and SLC25A24 in colon cancer, we executed several cytological experiments in vitro. We selected one human colonic epithelial cell line (NCM406) and seven colon cancer cell lines (DLD1, HCT116, HCT8, HT29, RKO, SW480, and SW620), examined the background expression level by RT-qPCR or western blotting, and recognized that both were expressed at relatively low levels in HCT-116 and RKO cells (Figs. 7A, S6A). To construct overexpression cell lines, we transfected lentiviral SLC25A5- and SLC25A24-overexpressing plasmids, transduced them into HCT-116 and RKO cells, and determined the success of upregulation (Figs. 7B, S6B). We evaluated the cell proliferation level post transfection of SLC25A5 and SLC25A24 by CCK-8 assay (Figs. 7C, S6C) and colony formation assay (Figs. 7D, S6D) and found that overexpression of SLC25A5, instead of SLC25A24, resulted in inhibition of tumorigenesis capacity. Staining for EdU (Fig. 7E) and TUNEL (Fig. 7F) and their quantification (Fig. 7G, H) indicated cell proliferation and apoptosis. The expression of programmed cell death-related biomarkers, such as Caspase-1, IL-1β, IL-18, and GSDMD, increased after SLC25A5 upregulation (Fig. 7I).

### Validation of the GSEA results of SLC25A5 with respect to glycolysis, lipid metabolism, and cancer-related cell signaling pathways

**Fig. 8:**
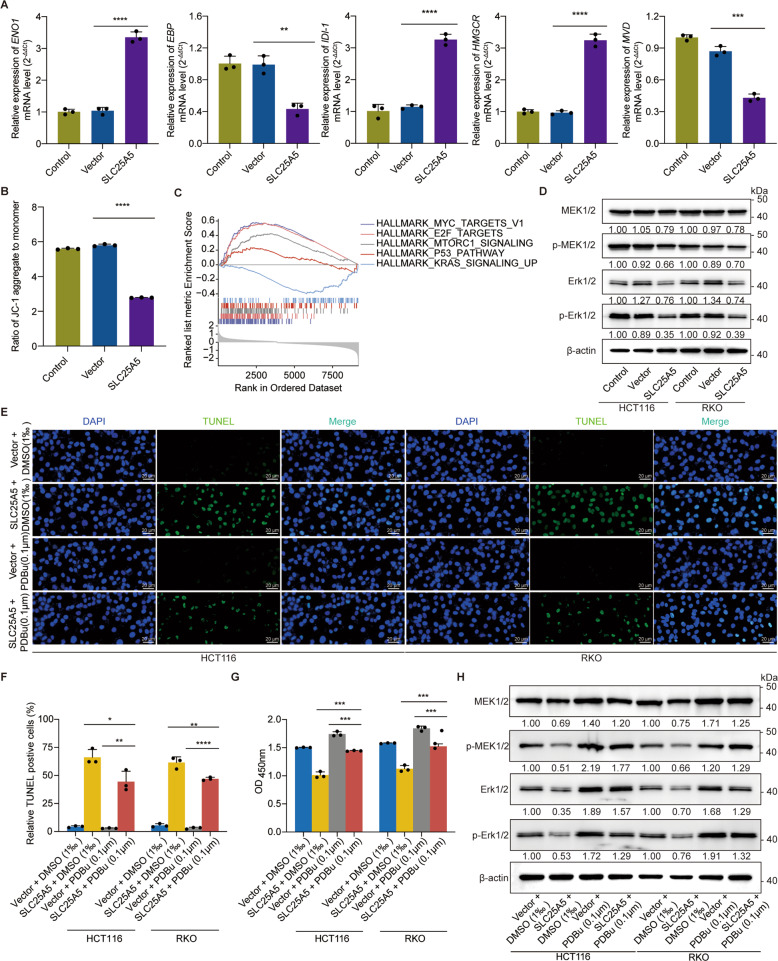
SLC25A5 was involved in mitochondrial metabolism and activity, while it exerted an effect on tumorigenesis via the MAPK signaling pathway. **A** The expression of ENO-1, IDI-1, and HMGCR was upregulated, while EBP and MVD were downregulated when SLC25A5 was overexpressed. **B** Decreased mitochondrial membrane potential was determined by JC-1 staining, suggesting the inhibition of mitochondrial activity. **C** GSEA hinted that high expression of SLC25A5 was negatively related to the KRAS signaling pathway. **D** Protein levels of MEK1/2, pMEK1/2, Erk1/2, and p-Erk1/2 were detected by western blotting. **E** Representative images of TUNEL staining in cells in the presence of the MAPK activator PDBu (0.1 μg for 24 h) or dimethyl sulfoxide (DMSO, 0.1‰) served as controls. with or without SLC25A5 overexpression. **F** Quantitative results of the TUNEL assay. **G** CCK8 assays for SLC25A5-overexpressing cells with or without 0.1 μg PDBu treatment. **H** Western blot analysis of MEK1/2, pMEK1/2, Erk1/2, and p-Erk1/2 in SLC25A5-overexpressing cells with or without 0.1 μg PDBu treatment. β-actin protein was used as the internal control. *n* = 3 biological replicates. Scale bar = 20 μm. Statistical analysis: Student’s t test. *p < 0.05, **p < 0.01, ***p < 0.001, ****p < 0.0001.

Based on the previous GSEA results, we found that glycolysis- and adipogenesis-related enzymes, such as ENO-1 (enolase 1), EBP (cholestenol delta isomerase), IDI-1 (isopentenyl-diphosphate delta isomerase 1), HMGCR (3-hydroxy-3-methylglutaryl-coenzyme A reductase), and MVD (mevalonate diphosphate decarboxylase), were differentially expressed when SLC25A5 (Fig. 8A) and SLC25A24 (Fig. S6E) were upregulated in HCT116 cells. Alterations in mitochondrial membrane potential (MMP) were detected by JC-1 staining (Figs. 8B, S6F). Since the HALLMARK_KRAS_SIGNALING_UP signature differed between SLC25A5 -low vs -high patients (Fig. 8C), the protein levels of the mitogen-activated protein kinase (MAPK) cascade, especially for p-MEK1/2 and p-Erk1/2, were downregulated (Fig. 8D). After treatment with phorbol-12,13-dibutyrate (PDBu, 0.1 μg) for 24 h in SLC25A5-overexpressing HCT116 and RKO cells, the activator of MAPK signaling pathway inhibited cell apoptosis (Fig. 8E, F) and promoted cell proliferation (Fig. 8G), suggesting the rescue of the SLC25A5 effect. The protein levels of p-MEK1/2 and p-Erk1/2 similarly increased in the presence of PDBu (Fig. 8H). These results indicated that SLC25A5 repressed tumorigenesis via the MAPK signaling pathway.

## Discussion

Although we have previously summarized the function, drug targets, and known inhibitors of solute carrier transporters across cancers [9], we are still interested in evaluating their potential use as diagnostic biomarkers or therapeutic targets for colon cancer.

There were 37 of 53 members of SLC25 presenting transcriptional differences. SLC25A3, SLC25A4, SLC25A5, SLC25A6, and SLC25A10 exhibited closer interactions than other members in the PPI network. SLC25A3 transports phosphate into the mitochondrial matrix and is highly expressed in chronic myeloid leukemia [10]. SLC25A4, SLC25A5, and SLC25A6 act as translocators of ADP from the cytoplasm into the mitochondrial matrix and of ATP from the mitochondrial matrix into the cytoplasm [11]. SLC25A4, which is downregulated in gastric cancer and upregulated in acute monocytic leukemia (AML) [12], was found to be a biomarker in renal cell carcinoma [13]. SLC25A6 induces apoptosis in human HeLa and HepG2 cells [14]. SLC25A10 transports small metabolites by exchanging dicarboxylates for phosphate, sulfate, and other small molecules and functions as an oncogene in osteosarcoma [15], lung cancer, and breast cancers [16].

Previous studies primarily focused on the DNA mutations of SLC25 and the pathogenesis of the diseases [17]. Ten mutations in SLC25A22, a glutamate carrier, have been identified as contributing to clinical symptoms related to dysfunction in the brain [18]. Although we found that ten of 37 DEMs presented high variation, the genetic changes in SLC25 did not appear to be relevant to the survival outcome of patients with colon cancer.

GDSC is an online public database for studying the connection between tumor mutation and cancer molecular therapy. GSK269962 is a selective ROCK (Rho-associated protein kinase) inhibitor, and GSK1363089-foretinib acts as a small molecule inhibitor of vascular endothelial growth factor and hepatocyte growth factor receptor tyrosine kinase family members. Trametinib and refametinib are potent, highly selective, and ATP noncompetitive inhibitors of MEK1 and MEK2. Although it may seem paradoxical, our results suggested that SLC25, and in particular, SLC25A5 and SLC25A24, might be involved in the EGFR and ERK-MAPK signaling pathways in colon cancer and potentially serve as treatment targets.

Consensus clustering, also named unsupervised clustering, is a common cancer subtype classification research method [19]. Hereby, we attempted to construct a different tumor classification system based on SCL25 DEMs. Due to sample size or clinical case heterogeneity, this analytic strategy could not determine an association between the mRNA expression levels and patient prognosis. Then, we adopted another algorithm strategy (rating the risk model in a two-step process) to quantify the contributions of DEMs to the prognosis of every case in the training set. We performed LASSO regression, which is commonly used for dimensionality reduction of transcriptome data in survival Cox regression models [20], and identified eight DEMs of SLC25 to construct a mathematical model. The satisfactory survival predictive ability of this risk model was shown in both the training (TCGA database) and validating (GEO database) datasets in patients with colon cancer.

SLC25A5 exhibited potential clinical predictive and prognostic value based on a series of bioinformatic analyses. SLC25A5 is named adenine nucleotide translocator 2 and presents as a gated pore exchanging ATP for ADP and is considered to have a correlation with glucose accumulation in various types of cancer [21]. Overexpression of SLC25A5 could both strengthen resistance to tyrosine kinase inhibitors in lung cancer [22] and reverse sorafenib resistance [23] via the PI3K/AKT pathway in hepatocellular carcinoma [24]. Although our study primarily reveals that SLC25A5 can repress cell growth by inhibiting the MAPK signaling pathway, whether SLC25A5 functions as a signal transduction regulator or exerts its biological function just by affecting the downstream metabolic network is unclear. Future insensitive research is required on this issue.

Song W summarized that solute carrier transporters regulate metabolic pathways and the levels of metabolites to mediate immune cell homeostasis [25]. Our results indicated a negative correlation between CD8 and SLC25A5, and the patients in the high-risk group showed higher immune infiltration and lower levels of glycolysis and cell apoptosis, which seemed contradictory to previous findings that increased tumor-infiltrating CD8^+^ lymphocytes were normally related to survival benefit in patients with cancer [26]. We deem the differences to lie in our clinical sample size and some yet unknown mechanisms. Jiang considered that dysfunction of T cells with a high level of infiltration or distinct exclusion of T cells from infiltrating tumors are two primary mechanisms resulting in tumor immune evasion [27], while Chang believed that the interaction of tumor metabolic reprogramming and the tumor microenvironment could drive the dysfunction of tumor-infiltrating T cells [28]. Our initial bioinformatic results need to be verified by truly in-depth experimental work.

Nevertheless, our study had some limitations. Since it was difficult to retrieve datasets that simultaneously included both transcriptomic and clinical survival data from the public databases, the small sample size of the TCGA row data and the shortcoming of the constructed riskScore model might introduce selection bias. A larger number of clinical cases and modification of the risk model are still required to evaluate the clinical correlations, such as tumor immune infiltration. Insights into the molecular mechanisms of SLC25A5 in mitochondrial metabolism and tumorigenesis should be elucidated by further laboratory work.

## Materials and methods

### Retrieval of raw data from public databases

Gene expression data based on RNA sequencing reported as transcripts per million (TPM), copy number variation (CNV), and clinical information of patients with colon cancer were obtained from the COAD (colon adenocarcinoma) project of TCGA (https://portal.gdc.cancer.gov/) as training sets. The data of alternative splicing events (ASEs) of the cases in the COAD project were obtained from the TCGASpliceSeq database (https://bioinformatics.mdanderson.org/TCGASpliceSeq/) [29]. The transcriptional expression data and clinicopathological information for the validation set were downloaded from the GSE17536 [30] and GSE39582 [31] sample sets from the GEO database (http://www.ncbi.nlm.nih.gov/geo) [32]. For comparison of unpaired samples, UCSC Cancer Browser UCSC Xena (https://xena.ucsc.edu/welcome-to-ucsc-xena/) was utilized to retrieve the RNA-seq data of colon cancer from TCGA and normal colon case sequencing data from The Genotype-Tissue Expression (GTEx) Project uniformly processed and unified by Toil Pipeline [33].

### Identification of DEMs, differentially expressed genes (DEGs), and coexpressed genes

The expression of SLC25 in colon cancers and normal colonic epithelium at the transcriptional level was investigated using the R limma package [34], and members with an absolute Log_2_ [Fold Change (FC)] value of more than 1 along with a *p* value less than 0.05 were identified as DEMs. The interaction between DEMs at the protein level was examined using STRING (http://string-db.org) [35]. The expression of DEMs was compared between the early-stage group (stages I and II) and the relatively advanced-stage group (stages III and IV) after all patients were regrouped and investigated in the ONCOMINE database (https://www.oncomine.org/resource/main.html) [36]. Statistically significant differences were determined using a Student’s T test or Wilcoxon rank-sum test, which was chosen based on whether the variable was continuous or discrete and whether a continuous variable was normally distributed or not. Survival analysis was performed using Kaplan–Meier curves. The search parameters on the ONCOMINE databases were set as follows: *p* value as 0.01, FC as 1.5, gene grade as 10%, and data type as mRNA. The expression of SLC25 in cancerous and normal tissue was also inspected at the protein level using an online tool available at The Human Protein Atlas (HPA) (http://www.proteinatlas.org) [37]. DEGs were determined with |Log_2_ [FC]| of more than 1 and *p* value of less than 0.05 by the R DESeq2 package (Version 1.26.0) [38]. Coexpressed genes were determined with a Spearman’s rank correlation test and the correlation coefficients were presented using *rho* value by the R Stat package (Version 3.6.3).

### Investigation of genetic variations

The genomic profiling, including DNA mutations and mRNA expression changes, of SLC25 was investigated using cBioPortal (http://www.cbioportal.org/) [39] with a z score threshold of ±2.0. Genes with a DNA mutation rate higher than 10% were examined, and their relationships with DFS and overall survival (OS) were determined using Kaplan–Meier curves. Statistical significance was determined using the log-rank test. The UpSetR [40] and RCircos packages [41] were utilized to plot the diagram of ASEs and CNV in 23 chromosome pairs for DEMs in patients with colon cancer.

### Survey of chemotherapeutics sensitivity

The transcriptome expression data of cell lines and the half maximal inhibitory concentration (IC_50_) of antitumor drugs were downloaded from the Genomics of Drug Sensitivity in Cancer (GDSC) database (https://www.cancerrxgene.org/) [42].

### Reclassification of colon cancer based on mitochondrial SLC25 family expression and construction of a risk model based on DEMs

A novel classification of colon cancer was established using DEMs of SLC25, and the ConsensusClusterPlus package [43] was used to determine the optimal number and stability among all categories. The value of new clusters on gene expression and clinical prognosis was evaluated using principal component and survival analyses. A two-step method was adopted to calculate the risk score. The cases obtained from the TCGA database were set as a training set, and DEMs were introduced into the model to identify genes related to prognosis using least absolute shrinkage and selection operator (LASSO) regression for dimension reduction. The candidates were entered into a multivariate Cox regression analysis for further selection to construct a risk model. The risk scoring formula was established by normalizing the gene expression, and weighing was performed using multivariate Cox coefficients.

### Functional clustering by enriching terms in Gene Ontology (GO) and signaling pathways in Kyoto Encyclopedia of Genes and Genomes (KEGG), gene set enrichment analysis (GSEA)

The R clusterProfiler package (Version 3.14.3) was used to perform enrichment of GO terms, KEGG pathways, and GSEA [44]. The R org.Hs.e.g.,.db package (Version 3.10.0) was used to convert symbols into Entrez IDs. The threshold conditions were set as an adjusted *p* value of less than 0.05 and a *q* value of less than 0.2. H.all.v7.2.symbols.gmt (Hallmarks) was considered a predefined gene set and downloaded from the Molecular Signatures Database (MSigDB, http://software.broadinstitute.org/gsea/msigdb). The gene set was considered significantly enriched if the false discovery rate (FDR) score and adjusted *p* value were less than 0.25 and 0.05, respectively. The R ggpolt2 package (Version 3.3.3) was used to visualize the results.

### Correlation analysis for immune cell infiltration, immune score, glycolysis, and apoptosis by the risk model

The use of the single-sample gene set enrichment analysis (ssGSEA) algorithm was based on the study of Bindea [45] and contained 29 gene sets labeling different tumor-infiltrating immune cell types. It was used to quantify the relative abundance of tumor-infiltrating immune cells in patients with colon cancer. The enrichment scores characterized the infiltration level of each type of immune cell in the samples and were calculated using ssGSEA in the R GSVA package (Version 1.34.0) [46]. The algorithm in the ESTIMATE package [47] quantified the immunological activity of gene expression profiles and produced the immune score of each tumor sample. After obtaining the gene set of glycolysis along with those of the apoptosis pathway from the KEGG pathway of GSEA [48], ssGSEA was executed to quantify glycolysis and apoptosis activities by scoring and evaluating the differences between the high- and low-risk groups.

### Construction and validation of a clinical prediction model

The risk scores were evaluated for predicting OS using univariate and multivariate Cox regression analyses. A nomogram was constructed for survival prediction in a training set of patients from TCGA and was validated in another set from GEO. Harrell’s consistency index (C-index) was measured to quantify the discrimination performances. A calibration curve was generated by comparing the predicted value from the nomogram with the actual survival to assess the performance of the nomogram. The survival package of R was used for survival analysis. Kaplan–Meier survival curves were used to show the difference in survival, and the log-rank test was used to evaluate the statistical significance of the differences between the two groups.

### Immunofluorescent localization in cells and validation in a tissue microarray containing colon cancer, adjacent normal tissues, and tumor-infiltrating immune cells using immunohistochemistry

SLC25 localization was detected by double immunofluorescence staining. COX IV (Abcam, Cambridge, MA, USA, ab202554) is a marker of mitochondria.

A total of 185 patients with colon cancer were pathologically diagnosed at Zhongshan Hospital Affiliated to Fudan University from October 2010 to January 2016. The median follow-up time was 37.8 months with an interquartile range of 20–60 months. Of these, 79 patients with progressive disease received radical surgical treatment, and 106 patients with advanced disease were administered systemic chemotherapeutic therapy. The recorded clinicopathological characteristics of the patients are listed in Tables S5 and S6. The patients were informed of the study and signed informed consent forms. Furthermore, the study was approved by the ethics committee of Zhongshan Hospital. Tissue microarray analysis was performed at the Department of Pathology under standard pathology operating procedures as follows: fresh tissues were fixed with 4% paraformaldehyde, embedded in paraffin, cut into sections, and placed onto glass slides. After the sections were deparaffinized, hydrophilized, and unmasked, the sections were blocked with bovine serum albumin, immunostained with primary antibodies against SLC25A5 (bs-9567R, 1:100, Bioss Biological Technology Co., Ltd, Beijing, China), SLC25A24 (bs-21226R, 1:100, Bioss Biological Technology Co., Ltd, Beijing, China), CD8 (GB13068, 1:100, Servicebio Technology Co., Ltd, Wuhan, China), MPO (GB11224, 1:500, Servicebio Technology Co., Ltd, Wuhan, China), CD19 (GB11061, 1:500, Servicebio Technology Co., Ltd, Wuhan, China), and CD68 (GB13067-M-2, 1:100, Servicebio Technology Co., Ltd, Wuhan, China) overnight at approximately 4 °C and subsequently incubated with a goat anti-rabbit secondary antibody (GB23303, 1:200; Servicebio Technology, Wuhan, China) for 30 min at approximately 20 °C. Then, the sections were stained with 3,3′-diaminobenzidine and counterstained with hematoxylin. Antigen-antibody complexes were detected using a panoramic slice scanner (3DHISTECH, Budapest, Hungary), recorded in a file, and viewed using CaseViewer 2.2 (3DHISTECH, Budapest, Hungary). The formula listed below was used to calculate the H-score and gene expression in the tissues using Quant Center 2.1 (3DHISTECH, Budapest, Hungary): H-SCORE = ∑ (PI × I) = (percentage of cells with weak intensity × 1) + (percentage of cells with moderate intensity × 2) + percentage of cells with strong intensity × 3). PI refers to the proportion of the positive signal pixel area, and I refers to the coloring intensity.

### Cell line culture, plasmid construction, lentivirus packaging, and cell transduction

A human normal colonic epithelial cell line (NCM460) and colon cancer cell lines (DLD1, HCT116, HCT8, HT29, RKO) were obtained from the Cell Bank of the Chinese Academy of Sciences (Shanghai, China) and cultured in Dulbecco’s modified Eagle’s medium (DMEM) (HyClone, Logan, UT, USA) containing 10% fetal bovine serum (FBS) (Gibco, Paisley, UK) in an incubator at 37 °C in a 5% CO_2_ air atmosphere. All cell lines were authenticated by short tandem repeat (STR) profiling (Genetic Testing Biotechnology Corporation, Suzhou, China) and routinely tested for mycoplasma using the MycoAlert™ Mycoplasma Detection Kit (Lonza; LT07-218, Rockland, ME, USA). The DNA coding regions (cDNAs) of SLC25A5 (NM_001152) and SLC25A24 (NM_013386) were synthesized and cloned into the CV061 vector at the XhoI/KpnI site. The constructed CV061-shSLC25A5 and CV061-shSLC25A24 were transfected into competent cells. HCT116 and RKO cells were infected with lentivirus containing SLC25A5-shRNAs, SLC25A24-shRNAs, and lenti-vector (NC) supernatants.

### RNA isolation and real-time quantitative polymerase chain reaction (RT-qPCR)

Total cellular RNA of cell lines was extracted with TRIzol reagent (Invitrogen, Carlsbad, CA, USA) and recombinant DNase I (Takara Bio, Beijing, China) according to the manufacturer’s recommendations. cDNA was generated with the PrimerScript™ RT master mix kit (Takara Bio, Dalian China). RT-qPCR was performed using the TB Green Premix Ex Taq™ kit (Takara Bio, Dalian China). The primer sequences of *SLC25A5*, *SLC25A24*, *ENO1*, *EBP*, *IDI-1*, *HMGCR*, *MVD,* and *GAPDH* used in the RT–qPCR are listed in Supplementary Table S7. All results were normalized to *GAPDH* mRNA expression and analyzed with the relative threshold cycle (Ct) method in the form of 2^−*ΔΔ*Ct^.

### Protein extraction and western blotting analysis

Protein was extracted using SDS lysis buffer (Beyotime Biotechnology, Shanghai, China), and equal amounts of total protein were separated by 10% SDS–PAGE and then transferred to nitrocellulose membranes (Millipore, USA). The membrane was blocked with 5% nonfat powdered milk (Sangon, China) in TBST at room temperature for one hour, incubated with primary antibodies overnight at 4 °C, and then washed with TBST followed by a 1 h incubation with secondary antibodies. The protein bands were visualized by an enhanced chemiluminescence detection kit (Tanon, China). Semiquantitative analysis of the protein density by western blotting was performed using ImageJ (Version 1.5.3). The following antibodies were used at a 1:1000 dilution: anti-SLC25A5 (Cat. #14671, Cell Signaling), anti-SLC25A24 (Cat. #221120, Abcam), anti-COX IV (Cat. #202554, Abcam), anti-phospho-MEK1/2 (Cat. #178876, Abcam), anti-MEK1/2 (Cat. #278564, Abcam), anti-phospho-ERK1/2 (Cat. #201015, Abcam), anti-ERK1/2 (Cat. #184699, Abcam), anti-caspase-1 (Cat. #207802, Abcam), anti-IL-1β (Cat. #254360, Abcam), anti-IL-18 (Cat. #243091, Abcam), anti-gasdermin D (Cat. #219800, Abcam), anti-β-actin (Cat. #4970, Abcam) and anti-GAPDH (Cat. #2118, Cell Signaling).

### Cell counting kit-8 (CCK-8) assay

The CCK-8 assay was performed to measure cell viability using a CCK-8 kit (Dojindo Laboratories, Kumamoto, Japan). Cells were seeded in 96-well plates at 1000 cells per well, and viable cells were detected every 24 h by measuring the absorbance at 450 nm after incubating for one hour at 37 °C with CCK-8 reagent. The experiment was replicated three times.

### Colony formation assay

For the colony formation assay, 1000 cells per well were seeded into a six-well plate and incubated for approximately 14 days. Colonies were considered to have proliferated over six generations. At the end of the experiments, colonies were washed with PBS, fixed in methanol for 15 min, and stained with 0.1% crystal violet solution for 30 min. The experiment was replicated three times, and the typical result was shown.

### 5-Ethynyl-2′-deoxyuridine (EdU) assays

For EdU staining, a Yefluor 594 EdU Imaging Kit (YEASEN Biotech Co. Ltd, Shanghai, China) was used according to the manufacturer’s protocol. HCT116 and RKO cells were seeded in 24-well plates at a density of 4 × 10^4^ cells/well for 24 h. The cell nuclei were stained using 4′,6-diamidino-2-phenylindole (DAPI) (Sigma-Aldrich, D9542). The positive cells (red fluorescence) were visualized by fluorescence microscopy, imaged, and analyzed using ImageJ software (Version 1.5.3). The experiment was replicated three times, and the typical result was shown.

### Terminal-deoxynucleotidyl transferase-mediated nick end labeling (TUNEL) assays

For TUNEL staining, Alexa Fluor 640 Lit (YEASEN, Shanghai, China) was used based on the supplier’s protocol. HCT116 and RKO cells were seeded in 24-well plates at a density of 1 × 10^4^ cells/well for 24 h. The cell nuclei were stained using DAPI (Sigma-Aldrich, D9542), and the positive cells (green fluorescence) were visualized by fluorescence microscopy and analyzed using ImageJ. The experiment was replicated three times, and the typical result was shown.

### Mitochondrial membrane potential (MMP) measurements

A JC-1 MMP Assay Kit (YEASEN, Shanghai, China) was used to detect MMP. HCT116 cells were seeded in 6-well plates at a density of 1 × 10^6^ cells/well for 24 h. JC-1 (0.5 ml, 10 μg/ml) staining solution was added to each well, and then the plates were incubated in a CO_2_ incubator for 15 min. After HCT116 cells were centrifuged at 1200 rpm for 5 min, washed twice with PBS and resuspended, fluorescence intensity values were detected using a multifunctional microplate reader (Spark, Tecan) and suggested as the ratio of JC-1 aggregate form to JC-1 monomer form. The experiment was replicated three times.

### Statistical analysis

Normality was estimated using the Shapiro–Wilk test of normality. The independent Student’s t test was used to estimate the statistical significance of differences between normally distributed continuous variables, whereas the Mann–Whitney U test (i.e., Wilcoxon rank-sum test) was used to estimate the statistical significance of differences between nonnormally distributed continuous variables. The chi-square test or Fisher’s exact test was used to study the statistical significance of differences between categorical variables. Spearman’s rank correlation analysis was performed to calculate correlation coefficients between two genes. Univariate and multivariate Cox regression analyses were performed to determine the independent prognostic factors. Variance was similar between the groups that are being statistically compared. The estimate of variation within each group of data was carried out by F-test. The pROC package of R was used to plot ROC curves [49] as well as to calculate the area under the curve (AUC) to assess the accuracy of the risk score and estimate the prognosis. A bilateral *p* value of less than 0.05 was considered statistically significant.

## Supplementary information

Supplementary Files

aj-checklist

## Data Availability

The raw data in the study can be retrieved from TCGA (The Cancer Genome Atlas) of COAD (colon adenocarcinoma) at https://portal.gdc.cancer.gov/, and GEO (Gene Expression Omnibus) at https://www.ncbi.nlm.nih.gov/geo/ of datasets GSE17536 and GSE39582. The authors declare that other data in this manuscript are available on request.

## Code availability

The raw data in the study can be retrieved from TCGA (The Cancer Genome Atlas) of COAD (colon adenocarcinoma) at https://portal.gdc.cancer.gov/, and GEO (Gene Expression Omnibus) at https://www.ncbi.nlm.nih.gov/geo/ of datasets GSE17536 and GSE39582. Other data in this manuscript are available on request.
