# Supplementary material for: An integrated bioinformatic investigation of mitochondrial solute carrier family 25 (SLC25) in colon cancer followed by preliminary validation of member 5 (SLC25A5) in tumorigenesis
Source: Cell Death Dis. 2022 Mar 14;13(3):237. doi: 10.1038/s41419-022-04692-1 (PMC8921248; doi:10.1038/s41419-022-04692-1)

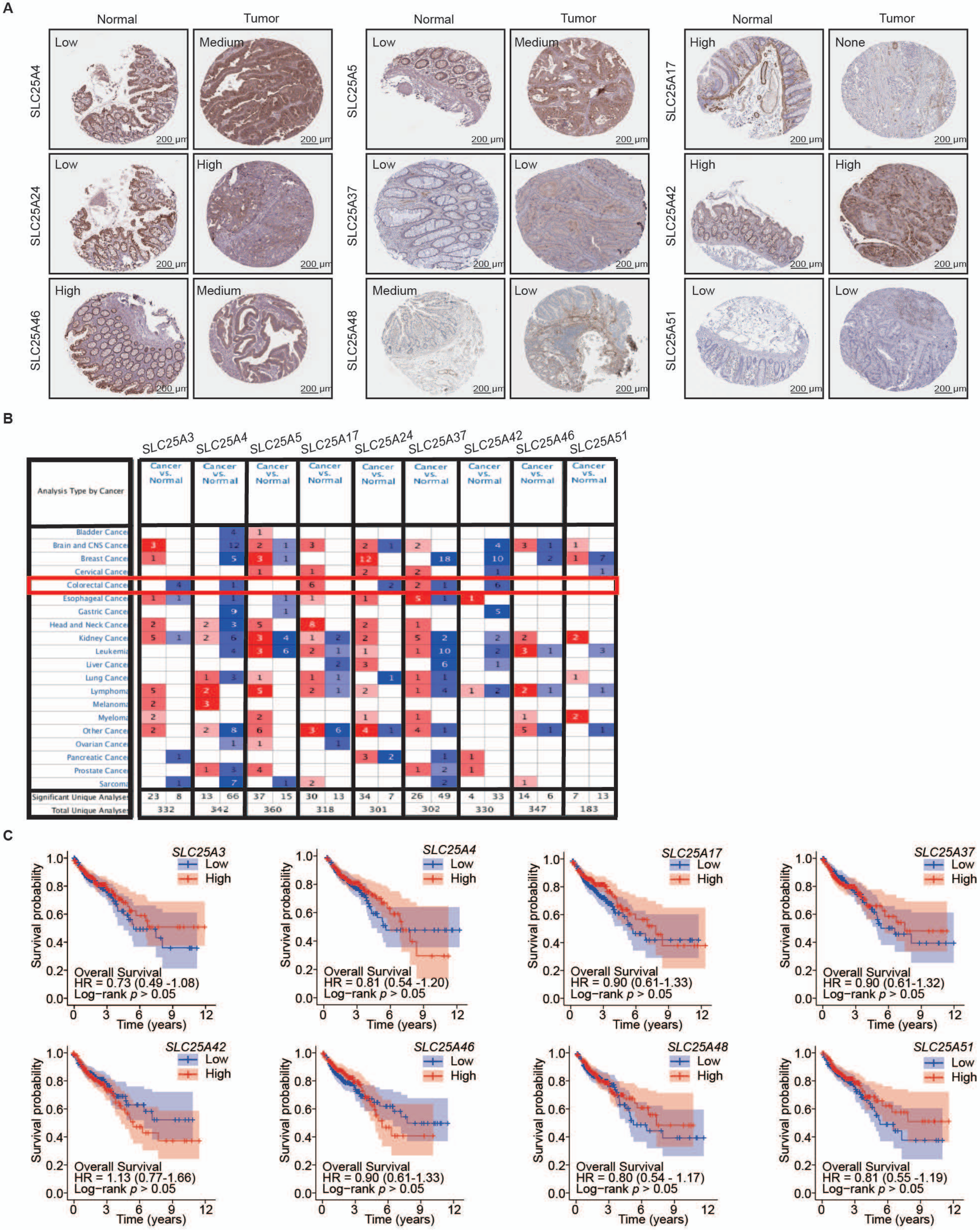
A
Normal
Tumor
SLC25A4
Low
Medium
SLC25A5
Low
Medium
SLC25A17
High
None
SLC25A24
Low
High
SLC25A37
Low
Low
SLC25A42
High
High
SLC25A46
High
Medium
SLC25A48
Medium
Low
SLC25A51
Low
Low
200 μm
B
SLC25A3
SLC25A4
SLC25A5
SLC25A17
SLC25A24
SLC25A37
SLC25A42
SLC25A46
SLC25A51
Analysis Type by Cancer
Cancer vs. Normal
Bladder Cancer
Brain and CNS Cancer
Breast Cancer
Cervical Cancer
Colorectal Cancer
Esophageal Cancer
Gastric Cancer
Head and Neck Cancer
Kidney Cancer
Leukemia
Liver Cancer
Lung Cancer
Lymphoma
Melanoma
Myeloma
Other Cancer
Ovarian Cancer
Pancreatic Cancer
Prostate Cancer
Sarcoma
Significant Unique Analyses
23 8
13 66
37 15
30 13
34 7
26 49
4 33
14 6
7 13
Total Unique Analyses
332
342
360
318
301
302
330
347
183
C
SLC25A3
HR = 0.73 (0.49 -1.08)
SLC25A4
HR = 0.81 (0.54 -1.20)
SLC25A17
HR = 0.90 (0.61-1.33)
SLC25A37
HR = 0.90 (0.61-1.32)
SLC25A42
HR = 1.13 (0.77-1.66)
SLC25A46
HR = 0.90 (0.61-1.33)
SLC25A48
HR = 0.80 (0.54 - 1.17)
SLC25A51
HR = 0.81 (0.55 -1.19)
Overall Survival
Log−rank p > 0.05
Low
High
Survival probability
Time (years)

Supplementary Fig. S1 Investigating the expression levels of DEMs in the Human Protein Atlas and ONCOMINE databases and clinical survival outcomes in TCGA. A Representative immunohistochemical images of mitochondrial SLC25 in cancer and normal tissues based on the Human Protein Atlas database. SLC25A4, SLC25A5, and SLC25A24 protein expression is relatively low in normal colon mucosa tissues and moderate or high in colon cancer tissues. SLC25A17 and SLC25A48 are moderately or highly expressed in normal tissues compared with their levels in cancerous tissues. SLC25A37 and SLC25A51 protein expression is low in both normal and cancerous tissues. SLC25A42 and SLC25A46 protein expression is moderate to high in both normal and cancerous tissues. **B** Transcriptional expression of representative members of mitochondrial SLC25 in 20 types of human cancer from the ONCOMINE database. **C** Kaplan-Meier curves suggested that patients with up- or downregulated DEMs experienced different survival times with no significant difference.

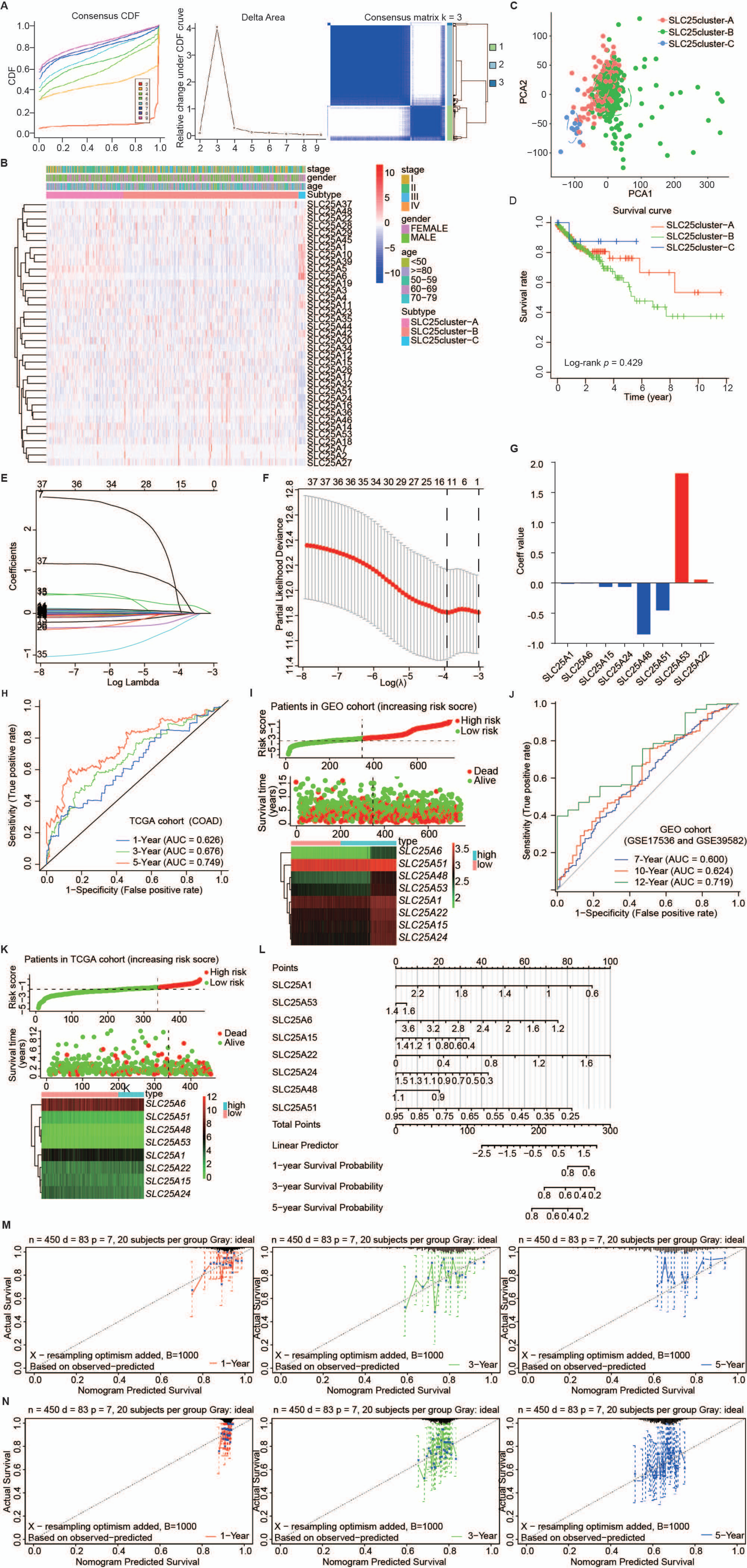

A
Consensus CDF
Delta Area
Consensus matrix k = 3
CDF
Relative change under CDF cruve
C
SLC25cluster-A
SLC25cluster-B
SLC25cluster-C
PCA1
PCA2
B
stage
gender
age
Subtype
FEMALE
MALE
SLC25cluster-A
SLC25cluster-B
SLC25cluster-C
D
Survival curve
Survival rate
Time (year)
Log-rank p = 0.429
E
Coefficients
Log Lambda
F
Partial Likelihood Deviance
Log(λ)
G
Coeff value
SLC25A1
SLC25A6
SLC25A15
SLC25A24
SLC25A48
SLC25A51
SLC25A53
SLC25A22
H
Sensitivity (True positive rate)
1-Specificity (False positive rate)
TCGA cohort (COAD)
1-Year (AUC = 0.626)
3-Year (AUC = 0.676)
5-Year (AUC = 0.749)
I
Patients in GEO cohort (increasing risk socre)
Risk score
High risk
Low risk
Survival time (years)
Dead
Alive
high
low
J
GEO cohort (GSE17536 and GSE39582)
7-Year (AUC = 0.600)
10-Year (AUC = 0.624)
12-Year (AUC = 0.719)
K
Patients in TCGA cohort (increasing risk socre)
L
Points
Total Points
Linear Predictor
1-year Survival Probability
3-year Survival Probability
5-year Survival Probability
M
n = 450 d = 83 p = 7, 20 subjects per group Gray: ideal
Actual Survival
Nomogram Predicted Survival
X - resampling optimism added, B=1000
Based on observed-predicted
1-Year
3-Year
5-Year
N

Supplementary Fig. S2 Risk score model constructed by LASSO regression combined with Cox regression. A Consensus matrix of cases from TCGA for k = 3. Clustering stability was examined using a one thousand iteration bootstrap. **B** Heatmap showing the differential expression of SLC25 and its relationship with clinicopathological characteristics. **C** Principal component analysis estimating the transcriptome landscape of the three clusters. **D** Survival analysis comparing the survival outcomes of the three clusters. **E** Results of LASSO regression analysis using 37 DEMs. **F** Thirteen of 37 DEMs were identified as the most valuable prognostic predictors. **G** Eight of 13 DEMs were included in the Cox proportional hazard model with their respective coefficient values. The risk score of each case from TCGA was subsequently calculated by these eight DEMs. **H** Time-dependent receiver operating characteristic curve (ROC) of 1-year, 3-year, and 5-year survival revealed that the model exhibited powerful efficacy in predicting the OS for cases in the training set. **I** Risk score distribution and gene expression pattern of the TCGA cohort. **J** Time-dependent ROC showed the predictive value of this model in GEO cohort. **J** Risk score distribution and gene expression pattern for the validation set. **L** A nomogram including 8 DEMs showed estimates of the prognosis for cases from the TCGA database. **M, N** The calibration curve suggested an optimal agreement between the estimated overall survival outcomes at 1 year, 3 years, and 5 years and the actual observed clinical outcomes of the cases from TCGA **(M)** and GEO datasets **(N)**.

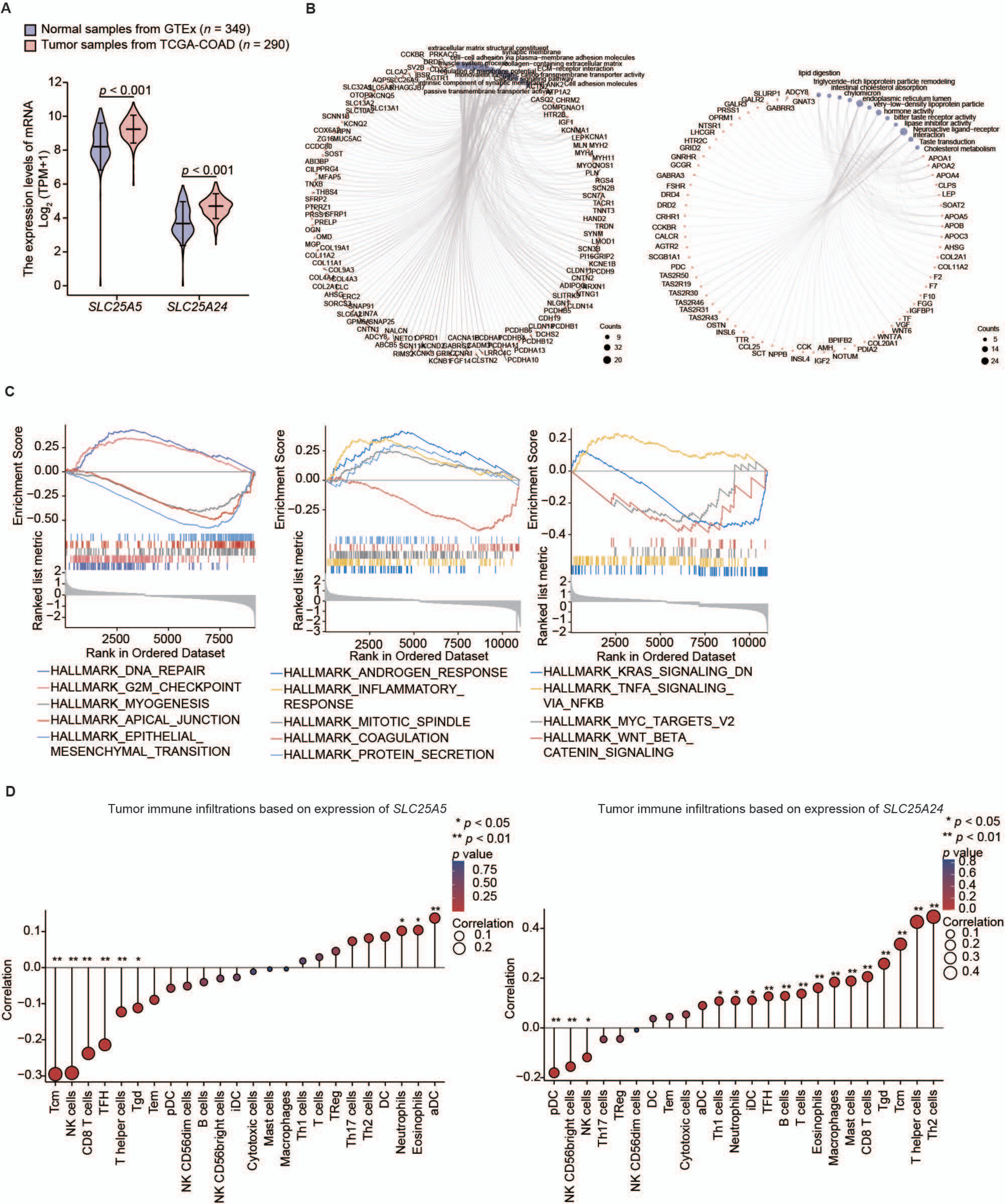
A
Normal samples from GTEx (n = 349)
Tumor samples from TCGA-COAD (n = 290)
The expression levels of mRNA Log2 (TPM+1)
p < 0.001
p < 0.001
SLC25A5
SLC25A24
B
Counts
C
Enrichment Score
Ranked list metric
Rank in Ordered Dataset
HALLMARK_DNA_REPAIR
HALLMARK_G2M_CHECKPOINT
HALLMARK_MYOGENESIS
HALLMARK_APICAL_JUNCTION
HALLMARK_EPITHELIAL_ MESENCHYMAL_TRANSITION
HALLMARK_ANDROGEN_RESPONSE
HALLMARK_INFLAMMATORY_ RESPONSE
HALLMARK_MITOTIC_SPINDLE
HALLMARK_COAGULATION
HALLMARK_PROTEIN_SECRETION
HALLMARK_KRAS_SIGNALING_DN
HALLMARK_TNFA_SIGNALING_ VIA_NFKB
HALLMARK_MYC_TARGETS_V2
HALLMARK_WNT_BETA_ CATENIN_SIGNALING
D
Tumor immune infiltrations based on expression of SLC25A5
Tumor immune infiltrations based on expression of SLC25A24
* p < 0.05
** p < 0.01
p value
Correlation
Tcm
NK cells
CD8 T cells
TFH
T helper cells
Tgd
Tem
pDC
NK CD56dim cells
B cells
NK CD56bright cells
iDC
Cytotoxic cells
Mast cells
Macrophages
Th1 cells
T cells
TReg
Th17 cells
Th2 cells
DC
Neutrophils
Eosinophils
aDC

Supplementary Fig. S3 Bioinformatic analyses of SLC25A5 and SLC25A24 based on data from TCGA. A The transcriptomic expression levels of SLC25A5 and SLC25A24 in samples from TCGA-COAD and The Genotype-Tissue Expression (GTEx). **B** Visualizing GO and KEGG enrichment analyses according to the expression of SLC25A5 (left) and SLC25A24 (right) in cycle diagrams. **C** Gene sets involved in cancer-related phenotypes and signaling pathways were enriched by different expression levels of SLC25A5 (left) and SLC25A24 (right). **D** Lollipop diagrams depict the relationship between the tumor immune infiltration of 23 common immune cells and the expression of SLC25A5 and SLC25A24.

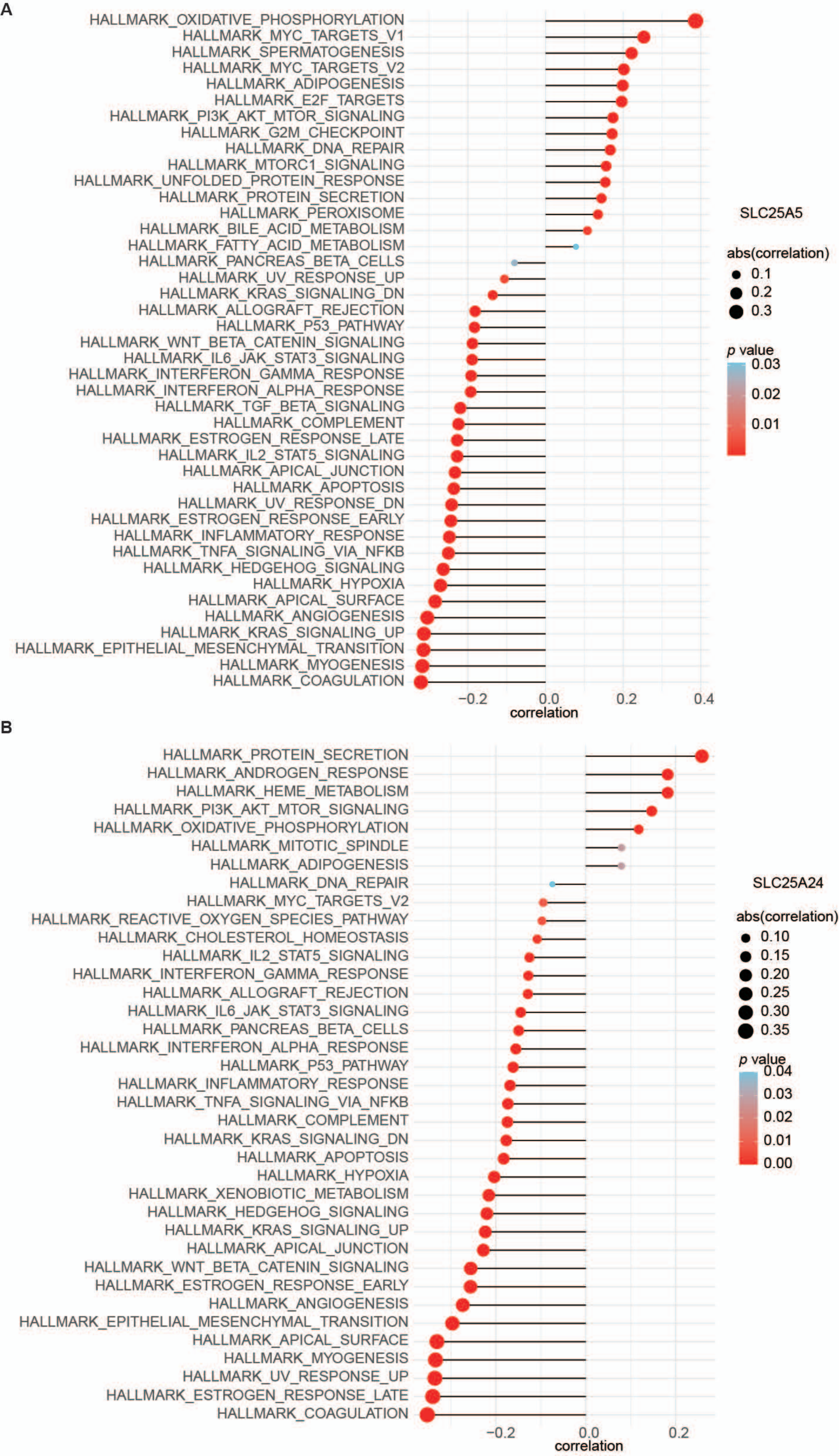
A
HALLMARK_OXIDATIVE_PHOSPHORYLATION
HALLMARK_MYC_TARGETS_V1
HALLMARK_SPERMATOGENESIS
HALLMARK_MYC_TARGETS_V2
HALLMARK_ADIPOGENESIS
HALLMARK_E2F_TARGETS
HALLMARK_PI3K_AKT_MTOR_SIGNALING
HALLMARK_G2M_CHECKPOINT
HALLMARK_DNA_REPAIR
HALLMARK_MTORC1_SIGNALING
HALLMARK_UNFOLDED_PROTEIN_RESPONSE
HALLMARK_PROTEIN_SECRETION
HALLMARK_PEROXISOME
HALLMARK_BILE_ACID_METABOLISM
HALLMARK_FATTY_ACID_METABOLISM
HALLMARK_PANCREAS_BETA_CELLS
HALLMARK_UV_RESPONSE_UP
HALLMARK_KRAS_SIGNALING_DN
HALLMARK_ALLOGRAFT_REJECTION
HALLMARK_P53_PATHWAY
HALLMARK_WNT_BETA_CATENIN_SIGNALING
HALLMARK_IL6_JAK_STAT3_SIGNALING
HALLMARK_INTERFERON_GAMMA_RESPONSE
HALLMARK_INTERFERON_ALPHA_RESPONSE
HALLMARK_TGF_BETA_SIGNALING
HALLMARK_COMPLEMENT
HALLMARK_ESTROGEN_RESPONSE_LATE
HALLMARK_IL2_STAT5_SIGNALING
HALLMARK_APICAL_JUNCTION
HALLMARK_APOPTOSIS
HALLMARK_UV_RESPONSE_DN
HALLMARK_ESTROGEN_RESPONSE_EARLY
HALLMARK_INFLAMMATORY_RESPONSE
HALLMARK_TNFA_SIGNALING_VIA_NFKB
HALLMARK_HEDGEHOG_SIGNALING
HALLMARK_HYPOXIA
HALLMARK_APICAL_SURFACE
HALLMARK_ANGIOGENESIS
HALLMARK_KRAS_SIGNALING_UP
HALLMARK_EPITHELIAL_MESENCHYMAL_TRANSITION
HALLMARK_MYOGENESIS
HALLMARK_COAGULATION
-0.2
0.0
0.2
0.4
correlation
SLC25A5
abs(correlation)
0.1
0.2
0.3
p value
0.03
0.02
0.01
B
HALLMARK_PROTEIN_SECRETION
HALLMARK_ANDROGEN_RESPONSE
HALLMARK_HEME_METABOLISM
HALLMARK_PI3K_AKT_MTOR_SIGNALING
HALLMARK_OXIDATIVE_PHOSPHORYLATION
HALLMARK_MITOTIC_SPINDLE
HALLMARK_ADIPOGENESIS
HALLMARK_DNA_REPAIR
HALLMARK_MYC_TARGETS_V2
HALLMARK_REACTIVE_OXYGEN_SPECIES_PATHWAY
HALLMARK_CHOLESTEROL_HOMEOSTASIS
HALLMARK_IL2_STAT5_SIGNALING
HALLMARK_INTERFERON_GAMMA_RESPONSE
HALLMARK_ALLOGRAFT_REJECTION
HALLMARK_IL6_JAK_STAT3_SIGNALING
HALLMARK_PANCREAS_BETA_CELLS
HALLMARK_INTERFERON_ALPHA_RESPONSE
HALLMARK_P53_PATHWAY
HALLMARK_INFLAMMATORY_RESPONSE
HALLMARK_TNFA_SIGNALING_VIA_NFKB
HALLMARK_COMPLEMENT
HALLMARK_KRAS_SIGNALING_DN
HALLMARK_APOPTOSIS
HALLMARK_HYPOXIA
HALLMARK_XENOBIOTIC_METABOLISM
HALLMARK_HEDGEHOG_SIGNALING
HALLMARK_KRAS_SIGNALING_UP
HALLMARK_APICAL_JUNCTION
HALLMARK_WNT_BETA_CATENIN_SIGNALING
HALLMARK_ESTROGEN_RESPONSE_EARLY
HALLMARK_ANGIOGENESIS
HALLMARK_EPITHELIAL_MESENCHYMAL_TRANSITION
HALLMARK_APICAL_SURFACE
HALLMARK_MYOGENESIS
HALLMARK_UV_RESPONSE_UP
HALLMARK_ESTROGEN_RESPONSE_LATE
HALLMARK_COAGULATION
-0.2
0.0
0.2
correlation
SLC25A24
abs(correlation)
0.10
0.15
0.20
0.25
0.30
0.35
p value
0.04
0.03
0.02
0.01
0.00

Supplementary Fig. S4 Lollipop plots suggested the correlation between members of SLC25 and hallmark sets of GSVA.

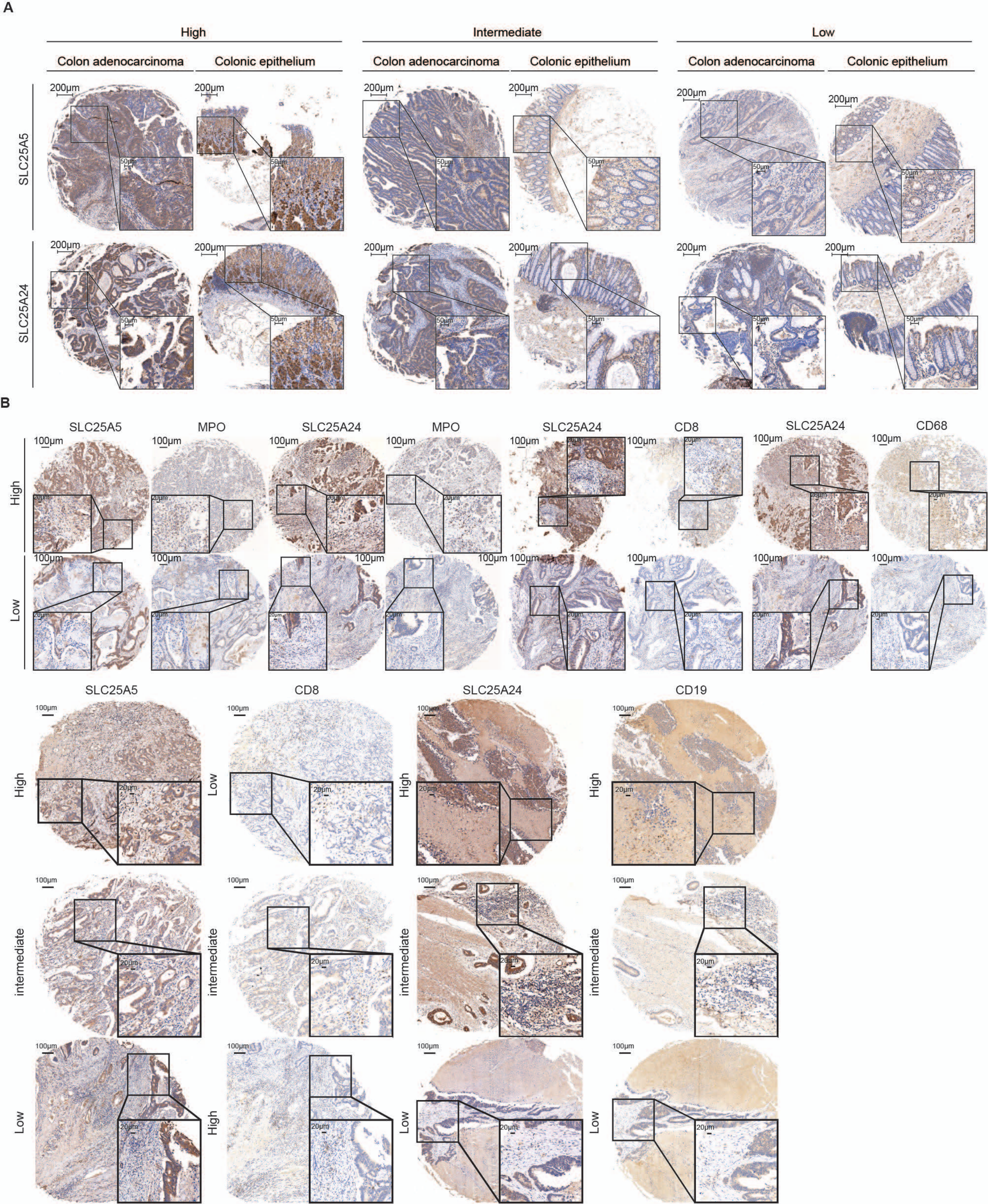
A
High
Intermediate
Low
Colon adenocarcinoma
Colonic epithelium
SLC25A5
SLC25A24
200μm
50μm
B
SLC25A5
MPO
SLC25A24
CD8
CD68
CD19
High
Low
intermediate
100μm
20μm

Supplementary Fig. S5 SLC25A5, SLC25A24, and biomarkers of infiltrating immune cells were immunohistochemically detected in colon cancer tissue samples. **A** Representative picture shows that SLC25A5 and SLC25A24 were expressed at high, moderate and low levels in colon adenocarcinoma or adjacent colonic epithelium. **B** Representative pictures show the expression of SLC25A5 and SLC25A24 in colon cancer and surrounding infiltrating immune cells, including neutrophils (MPO), CD8$^+$ T cells (CD8), and macrophages (CD68). Scale bars are 50 μm or 200 μm in **A** and 20 μm or 100 μm in **B**.

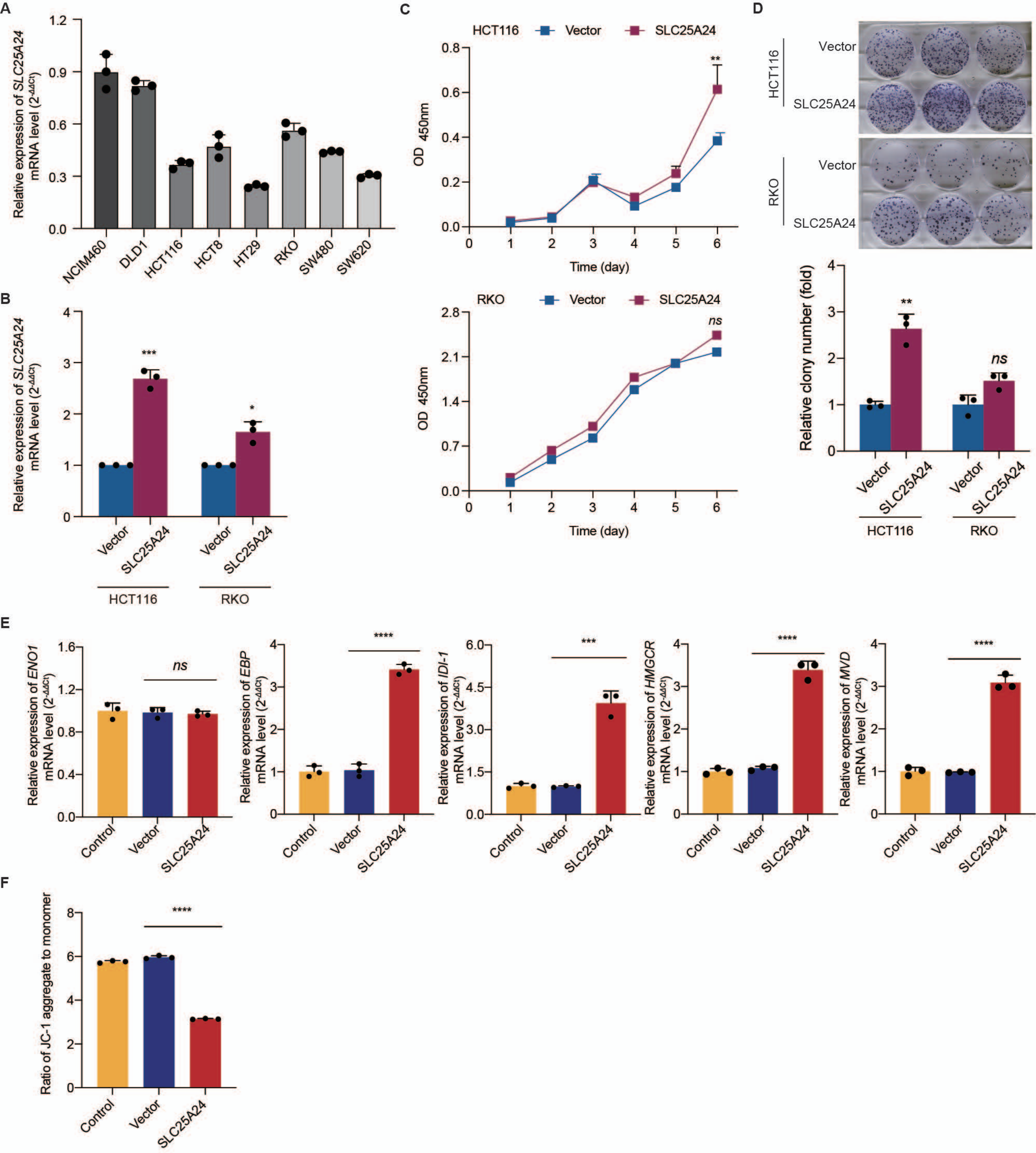
A
Relative expression of SLC25A24 mRNA level (2-ΔΔCt)
1.2
0.9
0.6
0.3
0.0
NCIM460
DLD1
HCT116
HCT8
HT29
RKO
SW480
SW620
B
Relative expression of SLC25A24 mRNA level (2-ΔΔCt)
4
3
2
1
0
***
*
Vector
SLC25A24
Vector
SLC25A24
HCT116
RKO
C
HCT116
Vector
SLC25A24
OD 450nm
0.8
0.6
0.4
0.2
0.0
**
0
1
2
3
4
5
6
Time (day)
RKO
Vector
SLC25A24
OD 450nm
2.8
2.1
1.4
0.7
0.0
ns
0
1
2
3
4
5
6
Time (day)
D
HCT116
Vector
SLC25A24
RKO
Vector
SLC25A24
Relative clony number (fold)
4
3
2
1
0
**
ns
Vector
SLC25A24
Vector
SLC25A24
HCT116
RKO
E
Relative expression of ENO1 mRNA level (2-ΔΔCt)
1.6
1.2
0.8
0.4
0.0
ns
Control
Vector
SLC25A24
Relative expression of EBP mRNA level (2-ΔΔCt)
4
3
2
1
0
****
Control
Vector
SLC25A24
Relative expression of IDI-1 mRNA level (2-ΔΔCt)
6.0
4.5
3.0
1.5
0.0
***
Control
Vector
SLC25A24
Relative expression of HMGCR mRNA level (2-ΔΔCt)
4
3
2
1
0
****
Control
Vector
SLC25A24
Relative expression of MVD mRNA level (2-ΔΔCt)
4
3
2
1
0
****
Control
Vector
SLC25A24
F
Ratio of JC-1 aggregate to monomer
8
6
4
2
0
****
Control
Vector
SLC25A24

Supplementary Fig. S6 The functional study of SLC25A24 in colon cancer. A The baseline expression of SLC25A24 at the mRNA level was tested**. B** SLC25A24 was overexpressed, and the expression level was tested. **C, D** The CCK8 assay **(C)** and colony formation assay **(D)** suggested that upregulation of *SLC25A24* tended to promote tumor growth; however, the difference did not reach statistical significance. **E** The expression levels of glycolysis- and adipogenesis-related genes when SLC25A24 was overexpressed. **F** Changes in mitochondrial membrane potential were determined by JC-1 staining assays. n = 3 biological replicates. Statistical analysis: Student' s t test. *ns*, no statistical significance; * $p < 0.05$, ** $p < 0.01$, *** $p < 0.001$.

Table S1. A brief list of metabolic substrates transported by SLC25.

Official Symbol	Aliases	Type of Substrates
SLC25A1	Citrate carrier, *CIC*	Di- and tri- carboxylates
SLC25A2	Ornithine carrier 2, *ORC-2*	Amino acids
SLC25A3	Phosphate carrier, *PiC*	Inorganic ions
SLC25A4	ADP/ATP carrier 1, *AAC-1*	Nucleotides
SLC25A5	ADP/ATP carrier 2, *AAC-2*	Nucleotides
SLC25A6	ADP/ATP carrier 3, *AAC-3*	Nucleotides
SLC25A7	Uncoupling protein 1, *UCP-1*	Protons
SLC25A8	Uncoupling protein 2, *UCP-2*	Protons
SLC25A9	Uncoupling protein 3, *UCP-3*	Protons
SLC25A10	Dicarboxylate carrier, DIC	Di- and tri- carboxylates
SLC25A11	Oxoglutarate carrier, OGC	Di- and tri- carboxylates
SLC25A12	Aspartate-glutamate carrier 1, AGC1	Amino acids
SLC25A13	Aspartate-glutamate carrier 2, AGC2	Amino acids
SLC25A14	Brain Mitochondrial Carrier Protein 1, *BMCP-1*; Uncoupling protein 5, UCP-5	Protons
SLC25A15	Ornithine carrier 1, *ORC-1*	Amino acids
SLC25A16	Graves' Disease Carrier Protein, *GDC*	Orphan transporters, unknown substrate
SLC25A17	Peroxisomal Membrane Protein, *PMP34*	Cofactors
SLC25A18	Glutamate carrier 2, *GC-2*	Amino acids
SLC25A19	Thiamine pyrophosphate carrier, *TPC*	Cofactors
SLC25A20	Carnitine-acylcarnitine carrier, *CAC*	Fatty acids
SLC25A21	Oxo dicarboxylate carrier, *ODC*	Di- and tri- carboxylates
SLC25A22	Glutamate carrier 1, *GC-1*	Amino acids
SLC25A23	ATP-Mg^{2+} / phosphate carrier 2, *APC-2*	Nucleotides
SLC25A24	ATP-Mg^{2+} / phosphate carrier 1, *APC-1*	Nucleotides

SLC25A25	ATP-Mg^{2+} / phosphate carrier 3, *APC-3*	Nucleotides
SLC25A26	S-adenosylmethionine carrier, *SAMC*	Cofactors
SLC25A27	Uncoupling protein 4, *UCP-4*	Protons
SLC25A28	Mitoferrin, *MTFRN*	Inorganic ions
SLC25A29	Basic amino acid carrier	Amino acids
SLC25A30	Kidney Mitochondrial Carrier Protein 1, *KMCP-1*	Orphan transporters, unknown substrate
SLC25A31	ADP/ATP carrier 4, *AAC-4*	Nucleotides
SLC25A32	Mitochondrial Folate Transporter/Carrier, *MFT*	Cofactors
SLC25A33	Pyrimidine Nucleotide Carrier 1, *PNC-1*	Nucleotides
SLC25A34	none	Orphan transporters, unknown substrate
SLC25A35	none	Orphan transporters, unknown substrate
SLC25A36	Pyrimidine Nucleotide Carrier 2, *PNC-2*	Nucleotides
SLC25A37	mitoferrin, MTFRN	Inorganic ions
SLC25A38	Glycine carrier, GLYC	Amino acids
SLC25A39	none	Orphan transporters, unknown substrate
SLC25A40	none	Orphan transporters, unknown substrate
SLC25A41	ATP-Mg^{2+} / phosphate carrier 4, *APC-4*	Nucleotides
SLC25A42	CoA and PAP carrier	Cofactors
SLC25A43	none	Orphan transporters, unknown substrate
SLC25A44	Branched-chain amino acid carrier	Amino acids
SLC25A45	none	Orphan transporters, unknown substrate
SLC25A46	none	Orphan transporters, unknown substrate
SLC25A47	Hepatocellular Carcinoma Down-Regulated Mitochondrial Carrier Protein, *HDMCP*	Protons
SLC25A48	none	Orphan transporters, unknown substrate
SLC25A49	Cell Proliferation-Inducing Protein 60, *PG60*	Orphan transporters, unknown substrate
SLC25A50	Mitochondrial Carrier Homolog 2, *MTCH-2*	Orphan transporters, unknown substrate

SLC25A51	Mitochondrial Nicotinamide Adenine Dinucleotide Transporter, Mitochondrial Carrier Triple Repeat 1, *MCART-1*	Nucleotides
SLC25A52	Mitochondrial Nicotinamide Adenine Dinucleotide Transporter, Mitochondrial Carrier Triple Repeat 2, *MCART-2*	Nucleotides
SLC25A53	Mitochondrial Carrier Triple Repeat 6, *MCART-6*	Orphan transporters, unknown substrate

2

Table S2. The baseline characteristics of cases from the colon cancer dataset (COAD) of The Cancer Genome Atlas (TCGA) database and a univariate analysis between subgroups based on a risk model constructed by mitochondrial solute carrier family 25 (SLC25).

**Variables**	**All (%)**	**Low risk (%)**	**High risk (%)**	***p* value**
Number	450 (100)	338 (100)	112 (100)	
Gender				0.620
Male	240 (53.3)	178 (52.7)	62 (55.4)	
Female	210 (46.7)	160 (47.3)	50 (44.6)	
Age				0.600
< 60	124 (27.6)	91 (26.9)	33 (29.5)	
>= 60	326 (72.4)	247 (73.1)	79 (70.5)	
T stage				0.280
Tis	1 (0.2)	1 (0.3)	0 (0.0)	
T1	10 (2.2)	7 (2.1)	3 (2.7)	
T2	80 (17.8)	67 (19.8)	13 (11.6)	
T3	305 (67.8)	225 (66.6)	80 (71.4)	
T4	54 (12.0)	38 (11.2)	16 (14.3)	
N stage				0.180
N0	270 (60.0)	211 (62.4)	59 (52.7)	
N1	102 (22.7)	73 (21.6)	29 (25.9)	
N2	78 (17.3)	54 (16.0)	24 (21.4)	
M stage				0.864
M0	333 (74.0)	251 (74.3)	82 (73.2)	
M1	60 (13.3)	45 (13.3)	15 (13.4)	
MX	50 (11.1)	36 (10.7)	14 (12.5)	
Pathologic stage				0.037
I and II	255 (56.7)	201 (59.5)	54 (48.2)	
III and IV	184 (40.9)	129 (38.2)	55 (49.1)	

Table S3. Thirty-seven differentially expressed members (DEMs) of mitochondrial SLC25 between colon cancer and corresponding paracancerous normal tissue in TCGA-COAD.

Member	Log_2 [Fold Change]	*p* value
SLC25A1	- 0.077	0.040
SLC25A2	2.046	0.001
SLC25A3	- 0.091	0.010
SLC25A4	- 0.665	0.000
SLC25A5	- 0.175	0.002
SLC25A6	0.574	0.000
SLC25A7	3.740	0.001
SLC25A10	0.603	0.000
SLC25A11	- 0.365	0.000
SLC25A12	- 0.382	0.000
SLC25A14	1.049	0.000
SLC25A15	1.025	0.000
SLC25A16	- 0.126	0.005
SLC25A17	0.703	0.000
SLC25A18	- 0.462	0.000
SLC25A19	0.712	0.000
SLC25A20	- 1.431	0.000
SLC25A22	1.159	0.000
SLC25A23	- 1.605	0.000
SLC25A24	- 0.554	0.000
SLC25A26	0.480	0.000
SLC25A27	1.550	0.000
SLC25A28	0.220	0.013
SLC25A29	1.438	0.000
SLC25A32	1.256	0.000
SLC25A34	- 3.943	0.000
SLC25A35	- 0.544	0.000
SLC25A36	0.654	0.000
SLC25A37	0.386	0.021
SLC25A39	0.772	0.000
SLC25A42	- 1.110	0.000
SLC25A44	- 0.249	0.000
SLC25A45	- 0.149	0.010
SLC25A46	- 0.142	0.001
SLC25A48	- 0.647	0.000
SLC25A51	0.295	0.000
SLC25A53	- 0.215	0.000

Table S4. Top five terms in GO and KEGG pathway enrichment analyses for SLC25A5 and SLC25A24 in colon cancer.

ONTOLOGY	ID	Description	GeneRatio	BgRatio	*p* value	*p*.adjust	*q*-value
**SLC25A5**							
BIOLOGICAL PROCESS	GO:0003012	muscle system process	32/358	465/18670	4.60e-10	1.16e-06	9.79e-07
BIOLOGICAL PROCESS	GO:0098742	cell–cell adhesion via plasma-membrane adhesion molecules	24/358	273/18670	6.20e-10	1.16e-06	9.79e-07
BIOLOGICAL PROCESS	GO:0042391	regulation of membrane potential	30/358	434/18670	1.49e-09	1.86e-06	1.57e-06
BIOLOGICAL PROCESS	GO:0006936	muscle contraction	26/358	360/18670	7.81e-09	7.30e-06	6.17e-06
BIOLOGICAL PROCESS	GO:0043062	extracellular structure organization	26/358	422/18670	1.90e-07	1.42e-04	1.20e-04
CELLULAR COMPONENT	GO:0097060	synaptic membrane	31/380	432/19717	3.70e-10	1.37e-07	1.04e-07
CELLULAR COMPONENT	GO:0062023	collagen-containing extracellular matrix	29/380	406/19717	1.55e-09	2.88e-07	2.18e-07
CELLULAR COMPONENT	GO:0099240	intrinsic component of synaptic membrane	18/380	164/19717	3.03e-09	3.74e-07	2.83e-07
CELLULAR COMPONENT	GO:0034703	cation channel complex	19/380	220/19717	5.75e-08	5.32e-06	4.02e-06
CELLULAR COMPONENT	GO:0042734	presynaptic membrane	16/380	161/19717	9.41e-08	6.96e-06	5.27e-06
MOLECULAR FUNCTION	GO:0005201	extracellular matrix structural constituent	18/353	163/17697	4.57e-09	2.41e-06	2.09e-06
MOLECULAR FUNCTION	GO:0015077	monovalent inorganic cation transmembrane transporter activity	26/353	382/17697	5.66e-08	1.49e-05	1.30e-05
MOLECULAR FUNCTION	GO:0022803	passive transmembrane transporter activity	28/353	457/17697	1.57e-07	2.76e-05	2.40e-05
MOLECULAR FUNCTION	GO:0015267	channel activity	27/353	456/17697	5.16e-07	6.80e-05	5.91e-05
MOLECULAR FUNCTION	GO:0046873	metal ion transmembrane transporter activity	26/353	438/17697	8.05e-07	8.48e-05	7.37e-05
KEGG PATHWAY	hsa04512	ECM-receptor interaction	9/155	88/8076	4.39e-05	0.008	0.007
KEGG PATHWAY	hsa04020	Calcium signaling pathway	13/155	201/8076	1.22e-04	0.008	0.007
KEGG PATHWAY	hsa04514	Cell adhesion molecules	11/155	149/8076	1.29e-04	0.008	0.007

KEGG PATHWAY	hsa04974	Protein digestion and absorption	9/155	103/8076	1.51e-04	0.008	0.007
KEGG PATHWAY	hsa04927	Cortisol synthesis and secretion	7/155	65/8076	2.29e-04	0.008	0.007
**SLC25A24**							
BIOLOGICAL PROCESS	GO:0044241	lipid digestion	7/311	19/18670	1.41e-08	4.38e-05	3.83e-05
BIOLOGICAL PROCESS	GO:0034370	triglyceride-rich lipoprotein particle remodeling	6/311	14/18670	5.46e-08	8.47e-05	7.41e-05
BIOLOGICAL PROCESS	GO:0030299	intestinal cholesterol absorption	6/311	15/18670	8.98e-08	9.28e-05	8.11e-05
BIOLOGICAL PROCESS	GO:0098856	intestinal lipid absorption	6/311	17/18670	2.16e-07	1.42e-04	1.24e-04
BIOLOGICAL PROCESS	GO:0034377	plasma lipoprotein particle assembly	7/311	28/18670	2.92e-07	1.42e-04	1.24e-04
CELLULAR COMPONENT	GO:0042627	chylomicron	6/335	13/19717	3.57e-08	1.09e-05	9.66e-06
CELLULAR COMPONENT	GO:0005788	endoplasmic reticulum lumen	21/335	309/19717	8.29e-08	1.26e-05	1.12e-05
CELLULAR COMPONENT	GO:0034361	very-low-density lipoprotein particle	6/335	20/19717	7.29e-07	5.56e-05	4.93e-05
CELLULAR COMPONENT	GO:0034385	triglyceride-rich plasma lipoprotein particle	6/335	20/19717	7.29e-07	5.56e-05	4.93e-05
CELLULAR COMPONENT	GO:0034364	high-density lipoprotein particle	6/335	26/19717	3.97e-06	2.42e-04	2.15e-04
MOLECULAR FUNCTION	GO:0005179	hormone activity	12/300	122/17697	1.11e-06	4.51e-04	3.96e-04
MOLECULAR FUNCTION	GO:0033038	bitter taste receptor activity	6/300	23/17697	1.79e-06	4.51e-04	3.96e-04
MOLECULAR FUNCTION	GO:0055102	lipase inhibitor activity	5/300	17/17697	7.09e-06	6.98e-04	6.13e-04
MOLECULAR FUNCTION	GO:0008527	taste receptor activity	6/300	29/17697	7.72e-06	6.98e-04	6.13e-04
MOLECULAR FUNCTION	GO:0048018	receptor ligand activity	23/300	482/17697	8.65e-06	6.98e-04	6.13e-04
KEGG PATHWAY	hsa04080	Neuroactive ligand–receptor interaction	24/138	341/8076	2.50e-09	4.82e-07	4.63e-07
KEGG PATHWAY	hsa04742	Taste transduction	9/138	86/8076	1.44e-05	0.001	0.001
KEGG PATHWAY	hsa04979	Cholesterol metabolism	6/138	50/8076	1.91e-04	0.012	0.012
KEGG PATHWAY	hsa04975	Fat digestion and absorption	5/138	43/8076	7.74e-04	0.037	0.036
KEGG PATHWAY	hsa05034	Alcoholism	10/138	187/8076	0.001	0.051	0.049

11

Table S5. The clinic-characteristics of 79 patients with progressive colon cancer and univariate analysis, including the expression of SLC25A5 and SCL25A24.

Variables	SLC25A5				SLC25A24			
	All cases (%)	High (%)	Low (%)	*p* value	All cases (%)	High (%)	Low (%)	*p* value
Number	72 (100)	34 (47.2)	38 (52.8)		78 (100)	43 (55.1)	35 (44.9)	
Gender				0.883				0.098
Male	43 (59.7)	20 (27.8)	23 (31.9)		48 (61.5)	30 (38.5)	18 (23.0)	
Female	29 (40.3)	14 (19.4)	15 (20.9)		30 (38.5)	13 (16.7)	17 (21.8)	
Age				0.598				0.294
< 60 years	32 (44.4)	14 (19.4)	18 (25.0)		35 (44.9)	17 (21.8)	18 (23.1)	
>= 60 years	4 (55.6)	20 (27.8)	20 (27.8)		43 (55.1)	26 (33.3)	17 (21.8)	
Tumor Site				0.471				0.255
Right	37 (51.4)	19 (26.4)	18 (25.0)		39 (50.0)	19 (24.4)	20 (25.6)	
Left	35 (48.6)	15 (20.8)	20 (27.8)		39 (50.0)	24 (30.8)	15 (19.2)	
Pathological Type				0.603				0.734
adenocarcinoma	53 (73.6)	26 (36.1)	27 (37.5)		55 (70.5)	31 (39.7)	24 (30.8)	
mucinous adenocarcinoma	19 (26.4)	8 (11.1)	11 (15.3)		23 (29.5)	12 (15.4)	11 (14.1)	
Pathological Grade				0.32				0.909
G1 and G2	51 (70.8)	26 (36.1)	25 (34.7)		54 (69.2)	30 (38.5)	24 (30.7)	
G3	21 (29.2)	8 (11.1)	13 (18.1)		24 (30.8)	13 (16.7)	11 (14.1)	
Tumor Size				0.067				0.235
< 5 cm	45 (62.5)	25 (34.7)	20 (27.8)		48 (61.5)	29 (37.2)	19 (24.3)	
>= 5 cm	27 (37.5)	9 (12.5)	18 (25.0)		30 (38.5)	14 (18.0)	16 (20.5)	
Gross Type				0.071				0.538
infiltrative	20 (27.8)	6 (8.3)	14 (19.5)		20 (25.6)	11 (14.1)	9 (11.5)	
elevated	25 (34.7)	16 (22.2)	9 (12.5)		27 (34.6)	15 (19.1)	16 (20.5)	
ulceration	27 (37.5)	12 (16.7)	15 (20.8)		31 (39.7)	17 (21.8)	10 (12.9)	

T Stage								
T1 and T2	12 (16.7)	7 (9.7)	5 (6.9)		14 (17.9)	9 (11.5)	5 (6.4)	
T3 and T4	60 (83.3)	27 (37.5)	33 (45.8)		64 (82.1)	34 (43.6)	30 (38.5)	
N Stage				0.185				0.001
N0	30 (41.7)	18 (25.0)	12 (16.7)		32 (41.0)	25 (32.0)	7 (9.0)	
N1	26 (36.1)	10 (13.9)	16 (22.2)		30 (38.5)	14 (17.9)	16 (20.6)	
N2	16 (22.2)	6 (8.3)	10 (13.9)		16 (20.5)	4 (5.1)	12 (15.4)	
Num of Lymph Nodes	72 (100)	1.35 ± 2.14	2.37 ± 2.63	0.076	78 (100)	1.27 ± 2.16	2.69 ± 2.74	0.016
Preoperative CEA Level	72 (100)	45.06 ± 118.50	96.04 ± 408.18	0.468	78 (100)	26.60 ± 101.87	113.80 ± 423.86	0.243
Preoperative CA199 Level	72 (100)	42.55 ± 50.64	45.74 ± 95.62	0.861	78 (100)	21.88 ± 28.98	70.54 ± 107.63	0.013
Preoperative CA125 Level	72 (100)	13.48 ± 11.23	20.58 ± 16.09	0.032	78 (100)	13.87 ± 9.37	20.69 ± 17.13	0.041
Preoperative AFP Level	72 (100)	2.96 ± 1.79	3.12 ± 1.31	0.671	78 (100)	3.20 ± 1.23	3.01 ± 1.80	0.591

Table S6. The clinic-characteristics of 106 patients with advanced colon cancer and tumor immune infiltrations.

Clinic-pathological characteristics	Number of Cases/Value	%
Primary tumor site		
Colon	63	59.4
Rectum	43	40.6
Colon location		
Right	34	32.1
Left	29	27.4
Gender		
Male	74	69.8
Female	32	30.2
Age		
less than 60	56	52.8
more than 60	50	47.2
Initial stage		
Advanced	54	50.9
Recurrence	52	49.1
Survival statues		
Death	50	47.2
Alive	56	52.8
Primary tumor resection		
Radical	54	50.9
Palliative	52	49.1
Anti-VEGF therapy		
Yes	52	49.1
No	54	50.9
Success staining		
SLC25A5	87	82.1
SLC25A24	103	97.2
CD8	98	92.5
MPO	92	86.8
CD19	94	88.7
CD68	101	95.3
Average H score		
SLC25A5	141.0	/
SLC25A24	156.2	/
CD8	9.3	/
MPO	36.3	/
CD19	42.2	/
CD68	24.1	/
Median H score		
SLC25A5	141.6	/
SLC25A24	161.4	/
CD8	4.3	/
MPO	35.6	/
CD19	31.7	/
CD68	19.8	/
Total	106	100

Table S7. The primer sequences for RT–qPCR in the study.

Species	Gene	Ensembl	Sequence (forward/reverse)
Human	*SLC25A5*	ENSG00000005022	5’ - TTATAGACTGCGTGGTCCGTA - 3’
			5’ - GGCGAAGTTAAGAGCCTGGG - 3’
	SLC25A24	ENSG00000085491	5’ - GGTGGCTTTCGACAGATGGTA - 3’
			5’ - GGTGGCTTTCGACAGATGGTA - 3’
	EBP	ENSG00000147155	5’ - ACTGTCCCTGTGCTGGTTTGC - 3’
			5’ - GGATGTATCGGCTGTCTCCCT - 3’
	IDI-1	ENSG00000067064	5’ - GCTAAGATTACCTTTCCAGGTT - 3’
			5’ - TCTGTGCTGCTCGCCTCACT - 3’
	ENO-1	ENSG00000074800	5’ - GCCTCCTGCTCAAAGTCAAC - 3’
			5’ - AACGATGAGACACCATGACG - 3’
	HMGCR	ENSG00000113161	5’ - AGGAGGCATTTGACAGCACT - 3’
			5’ - ACCTGGACTGGAAACGGATA - 3’
	MVD	ENSG00000167508	5’ - GCTGGCGGCAGTCACTTGTA - 3’
			5’ - TGCTGATGACGGCTGTTGTG - 3’
	GAPDH	ENSG00000111640	5’ - GACAGTCAGCCGCATCTTCT - 3’
			5’ - GCGCCCAATACGACCAAATC - 3’

Base line expression of SLC25A5

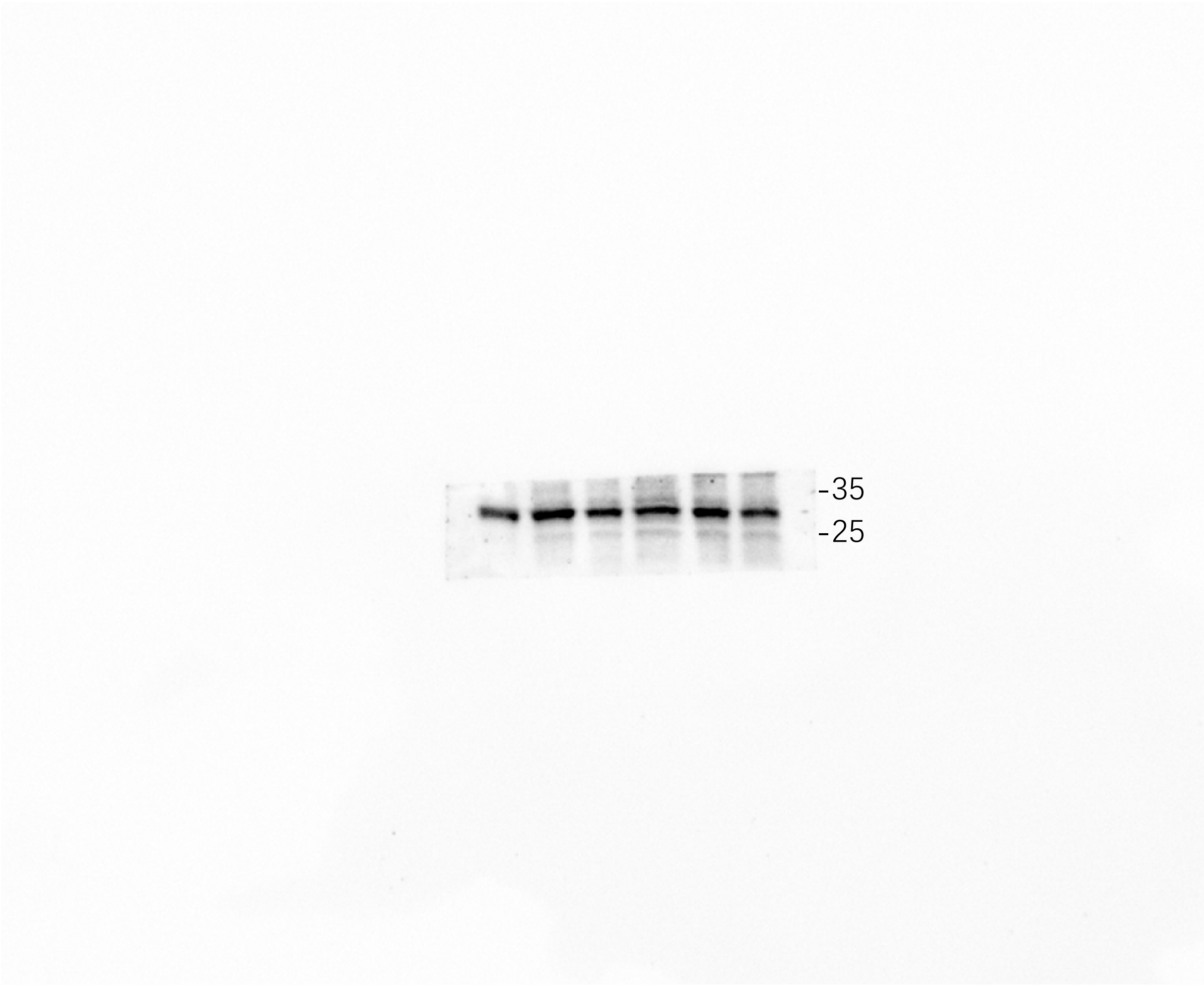

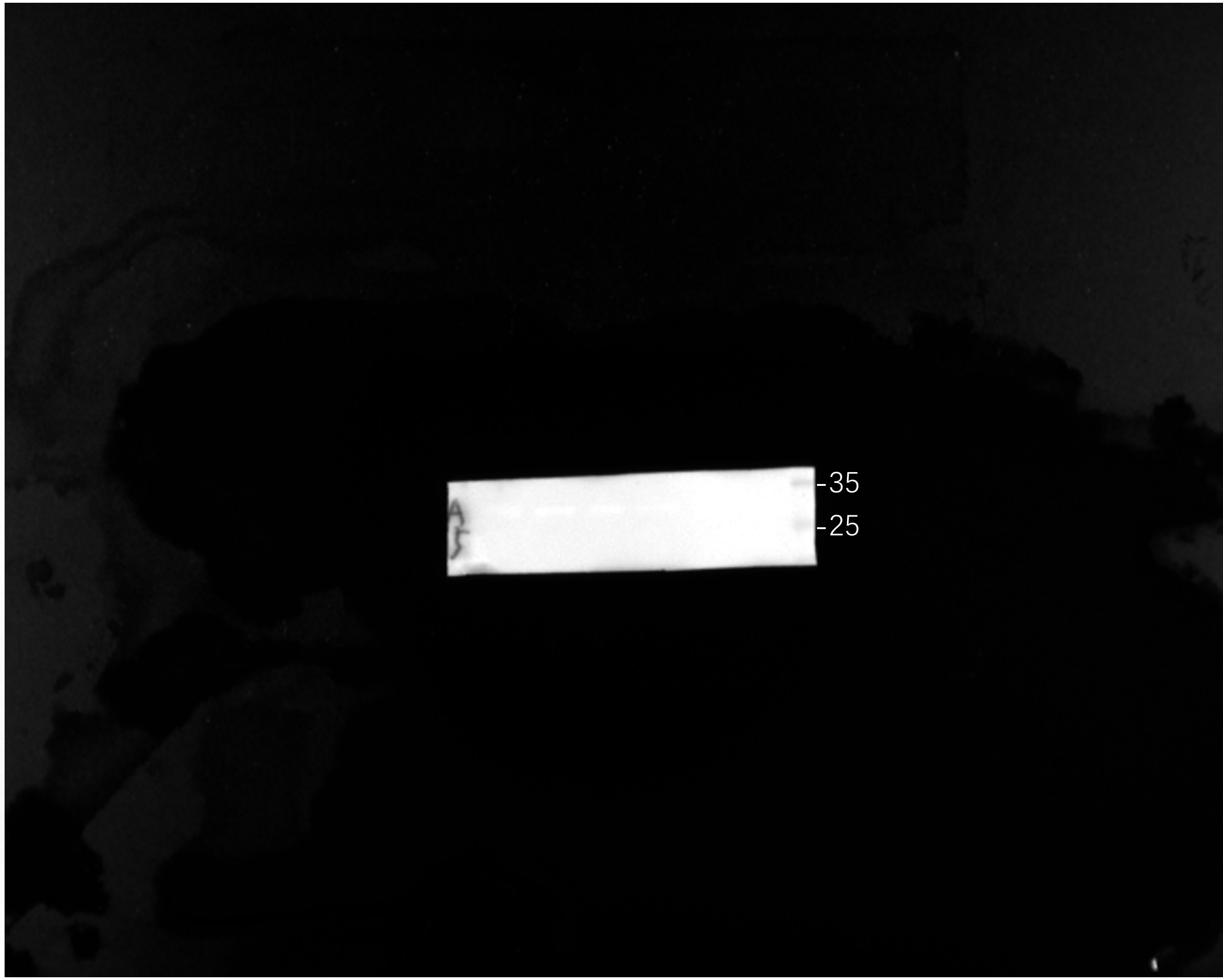

GAPDH of base line expression of SLC25A5

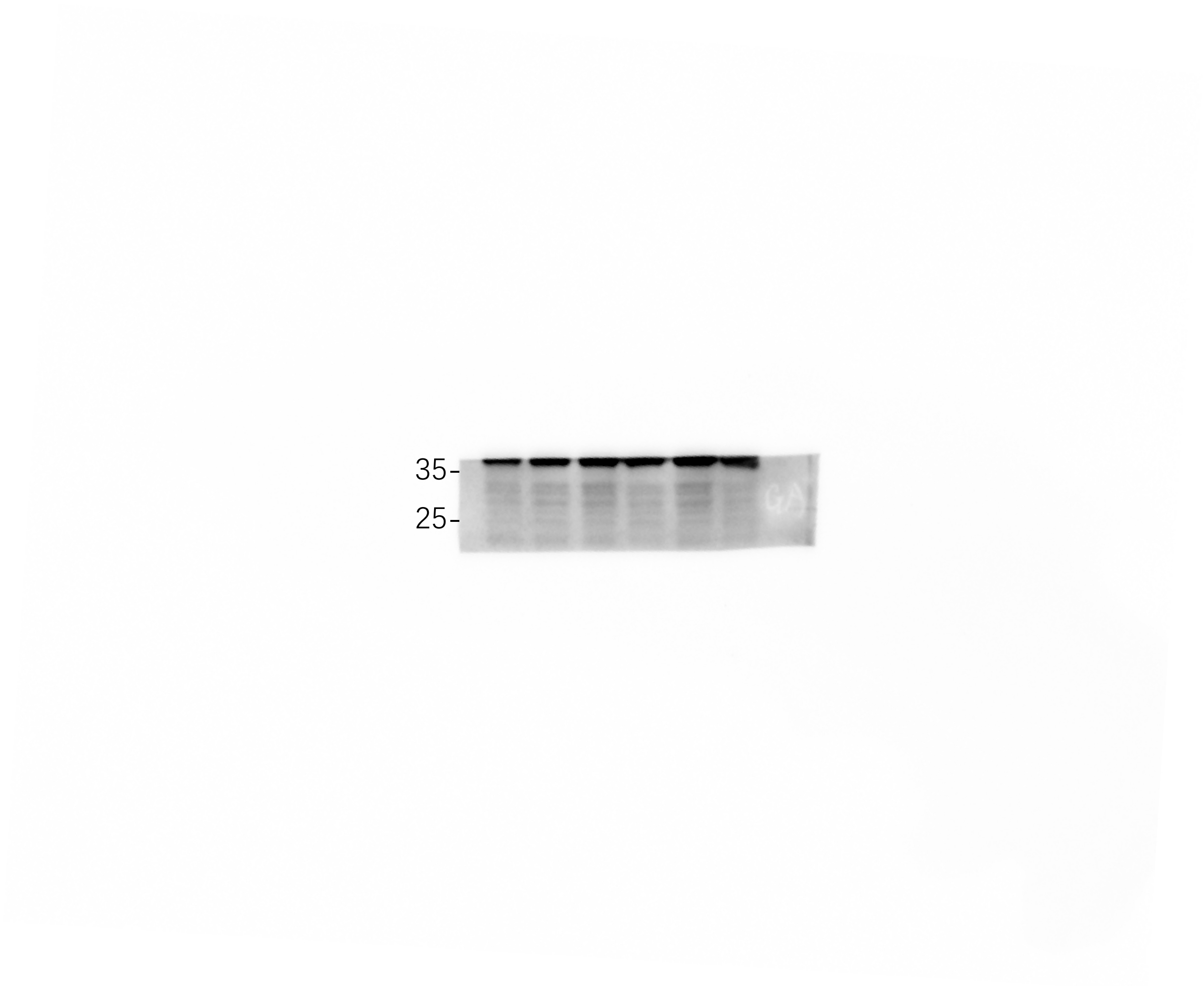

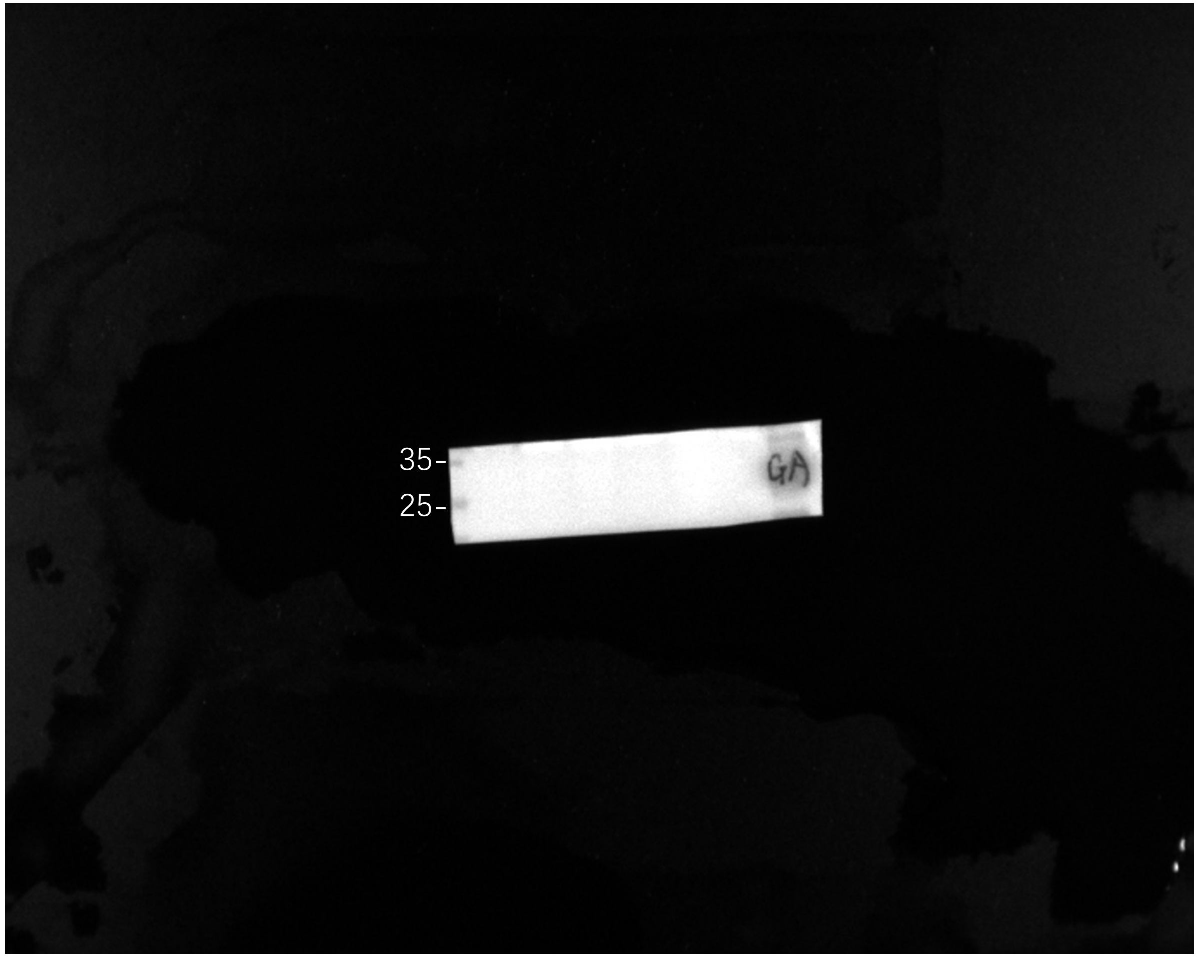

Overexpressing of SLC25A5

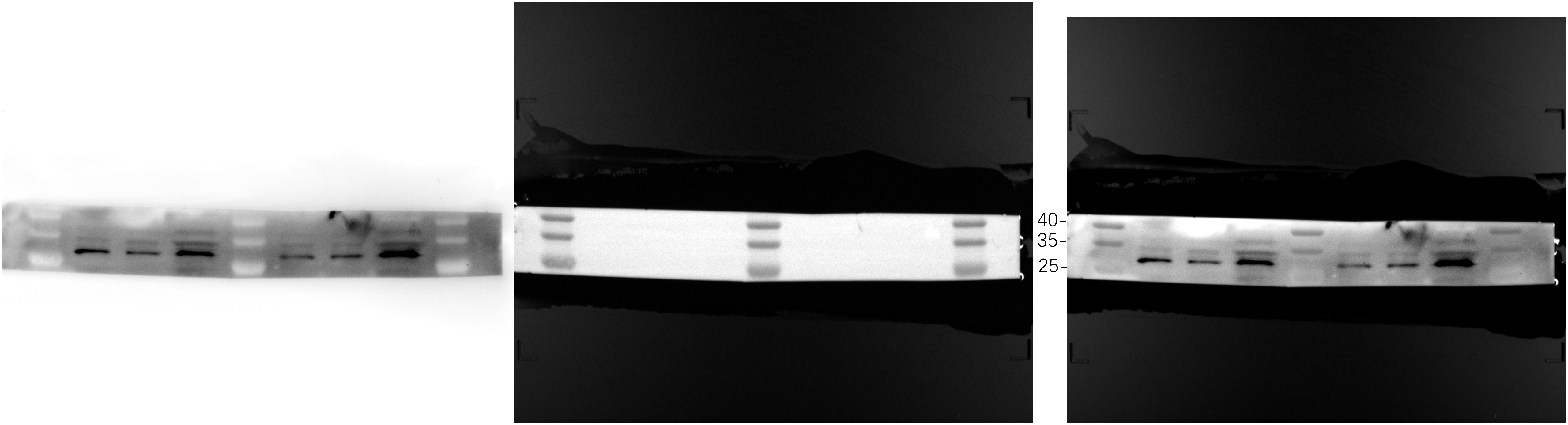

GAPDH of overexpressing of SLC25A5

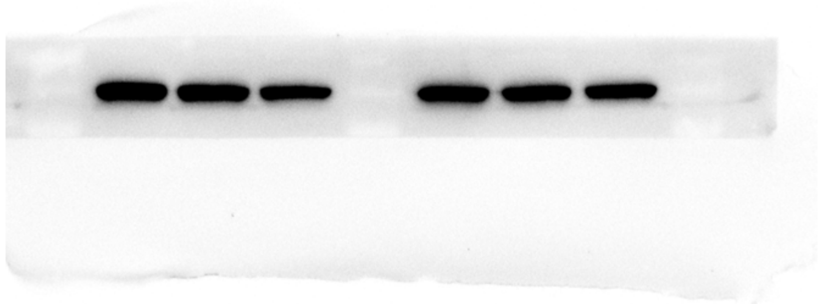

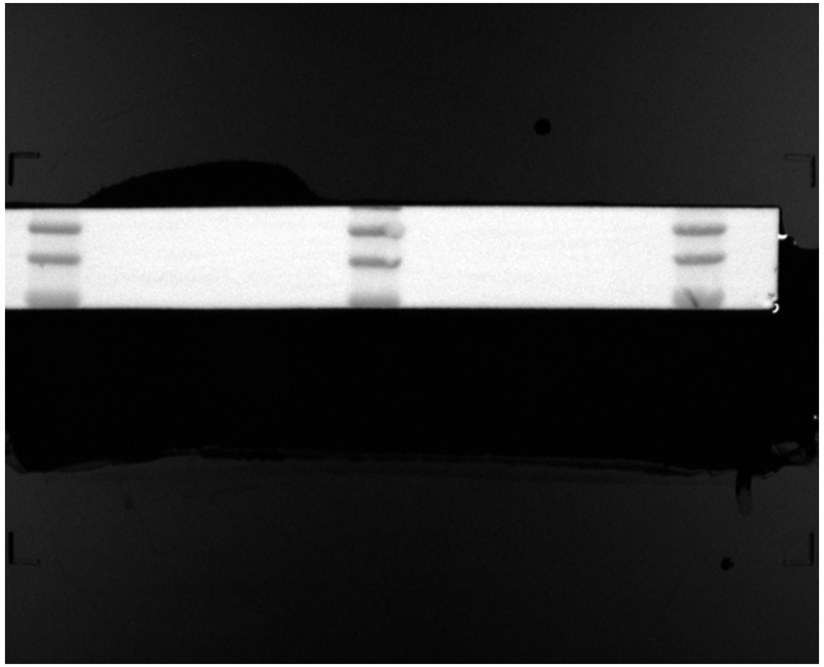

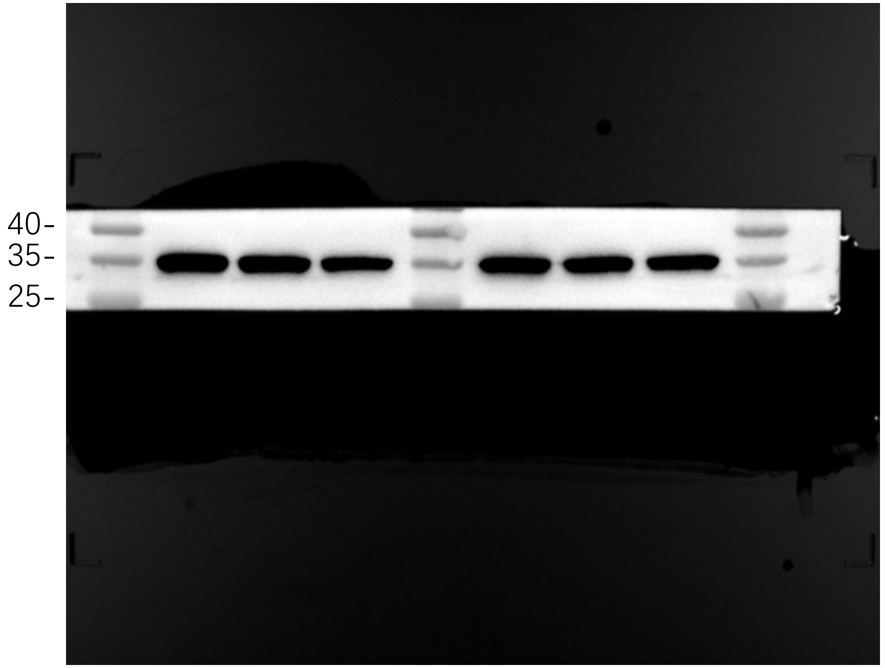

Caspase-1

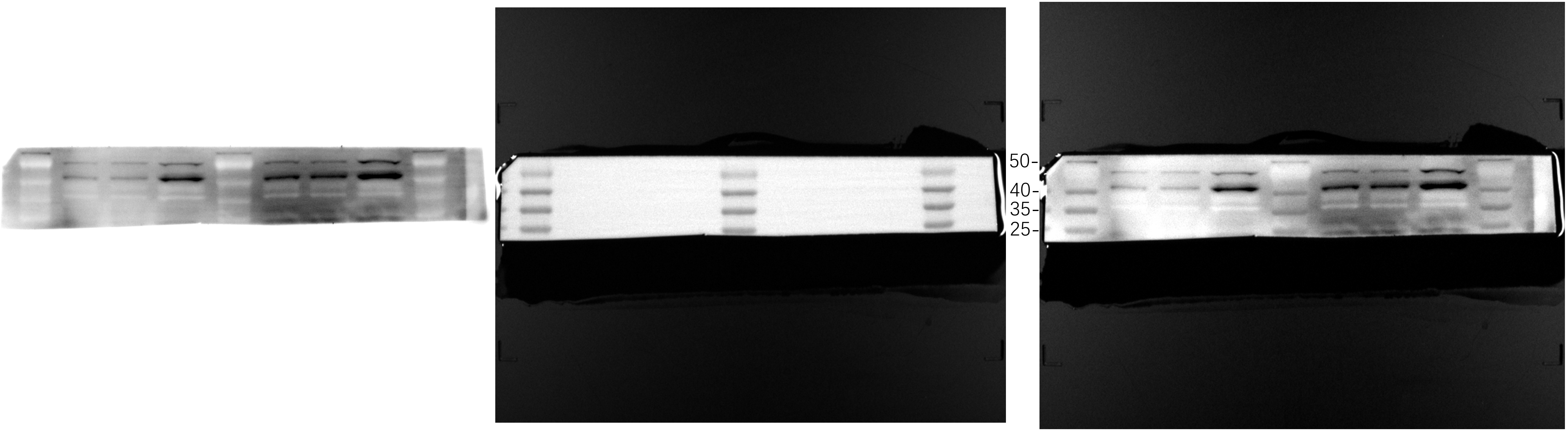

IL-1β

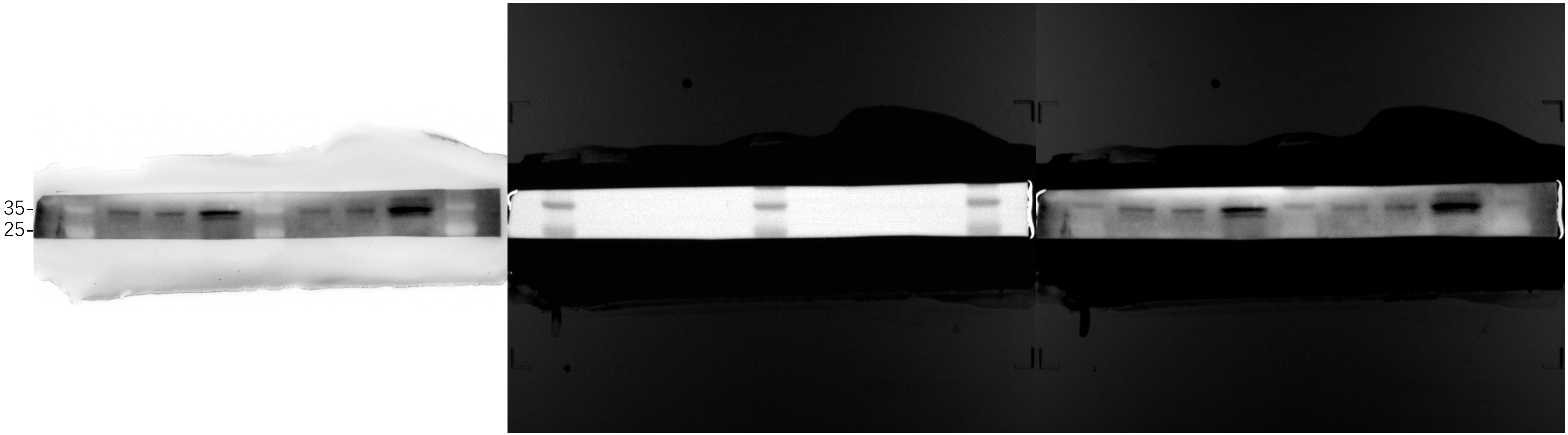

IL-18

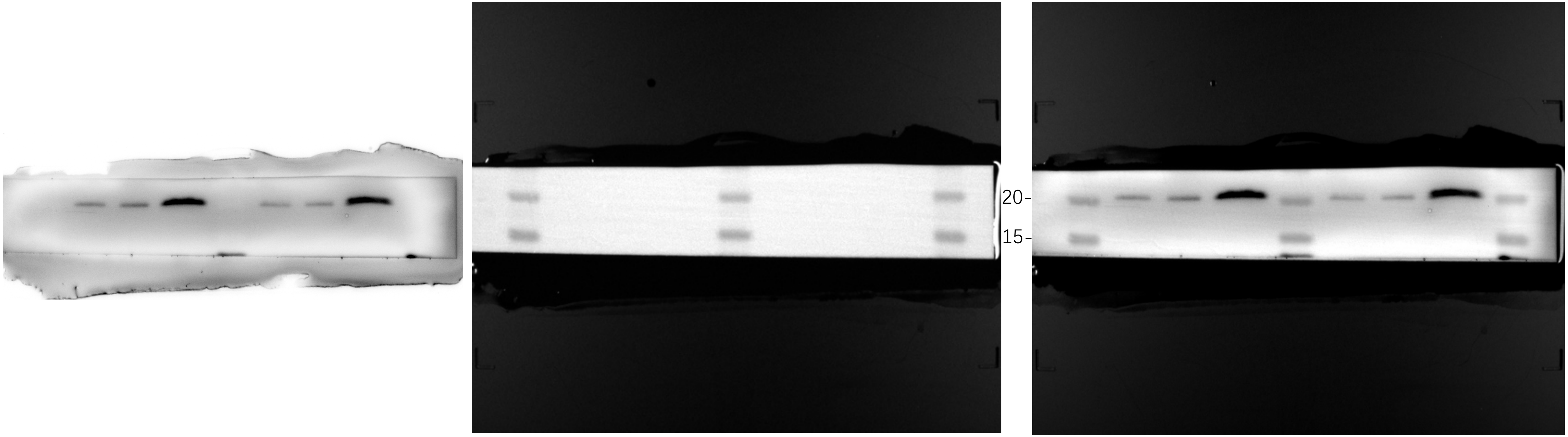

GSDMD

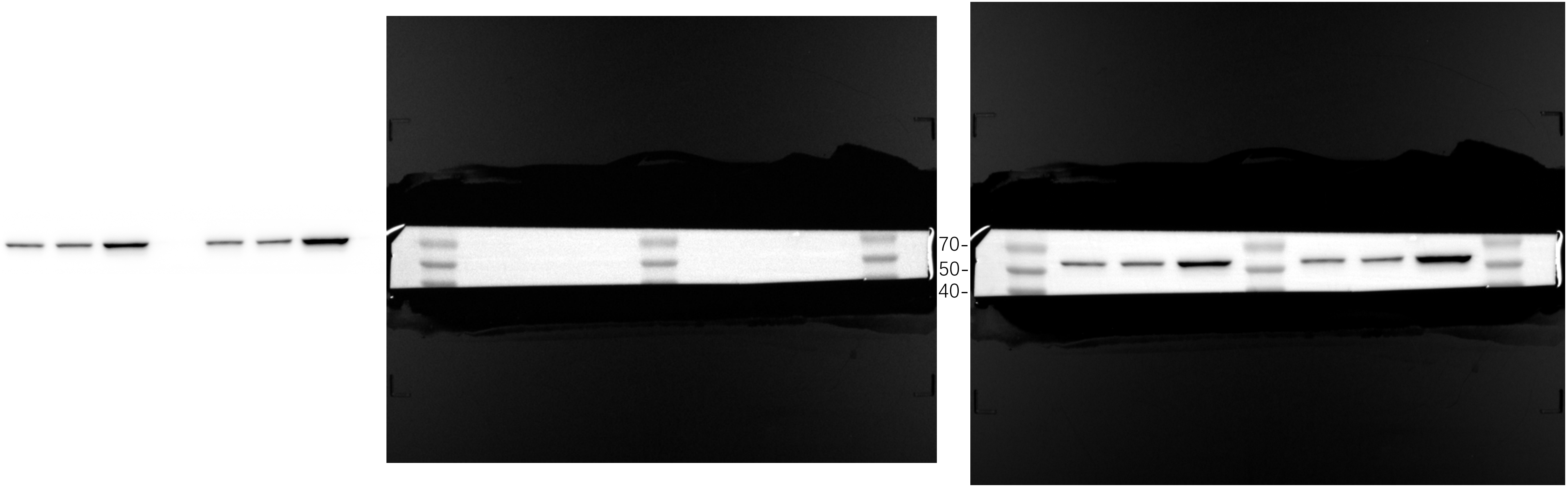

GAPDH

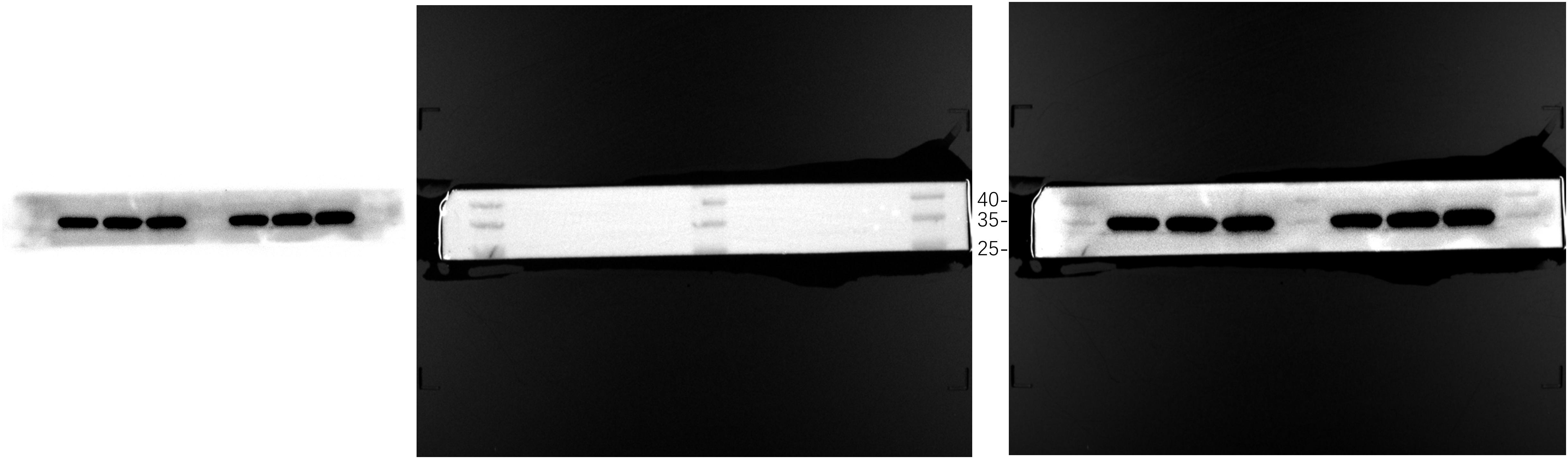

MEK1/2

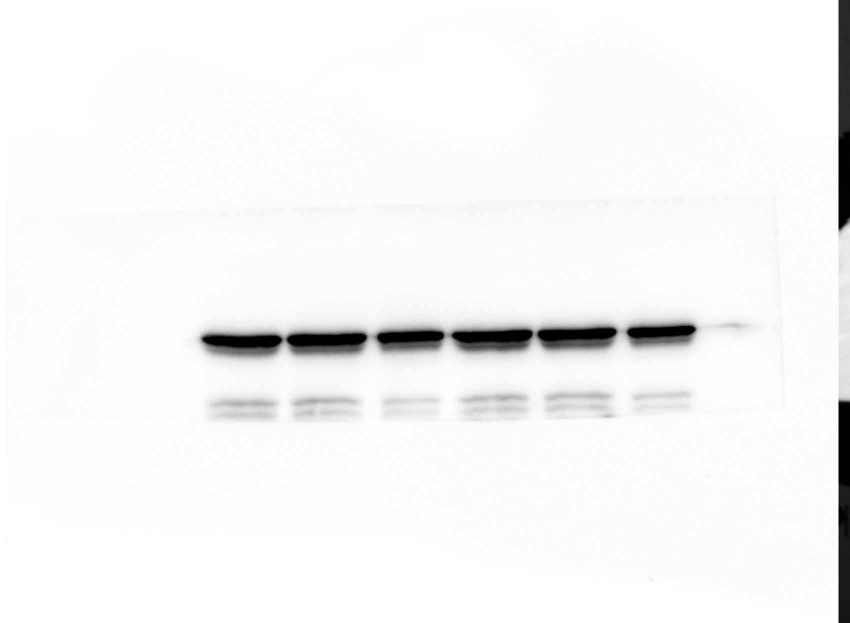

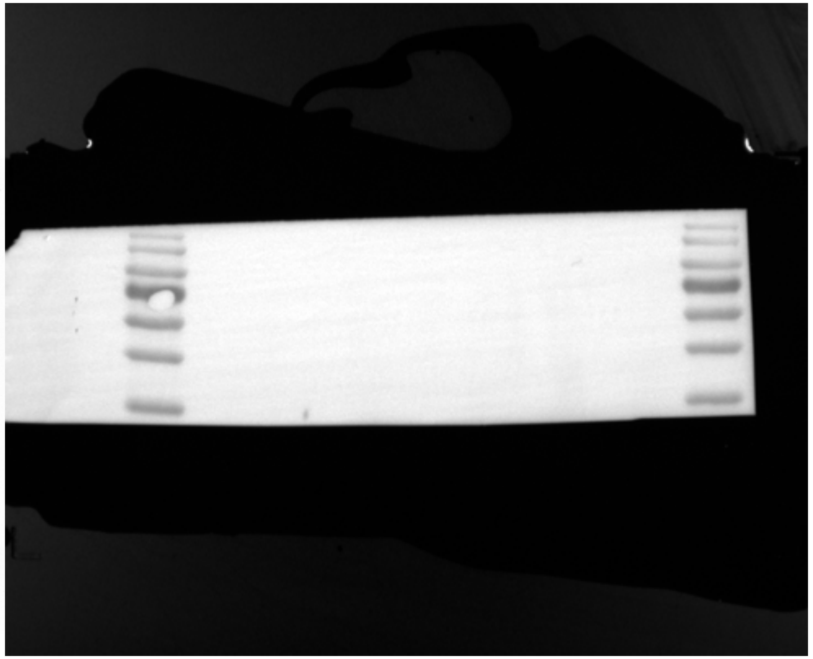

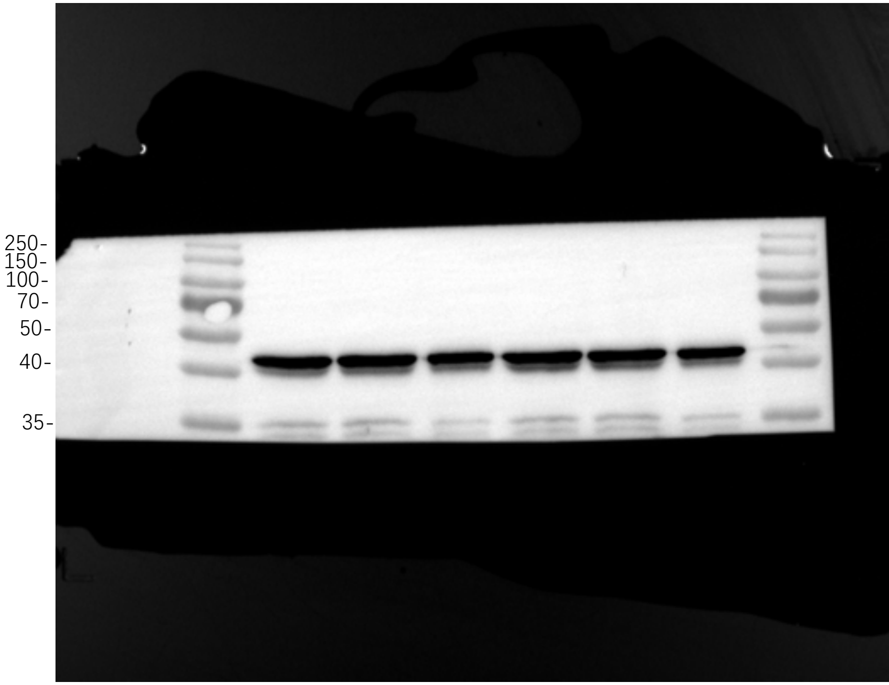

p-MEK1/2

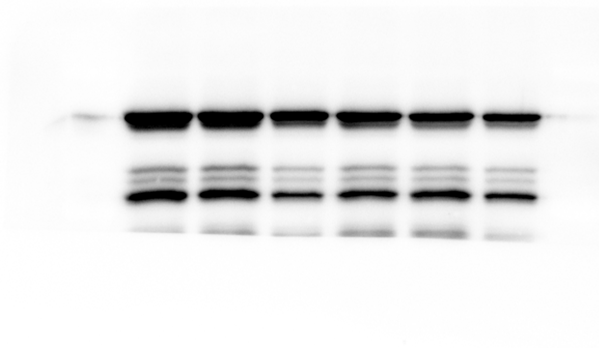

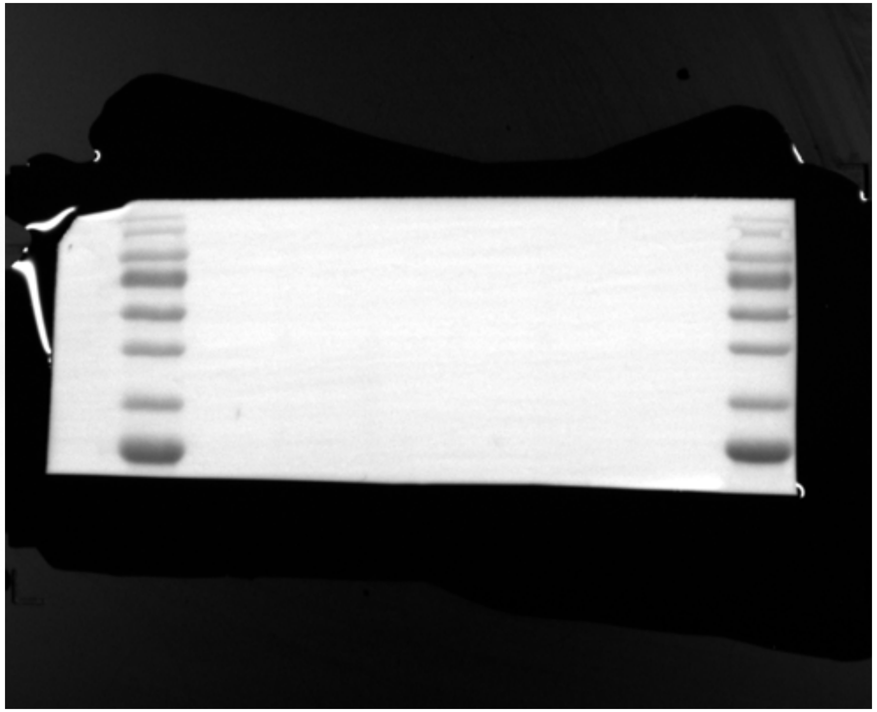

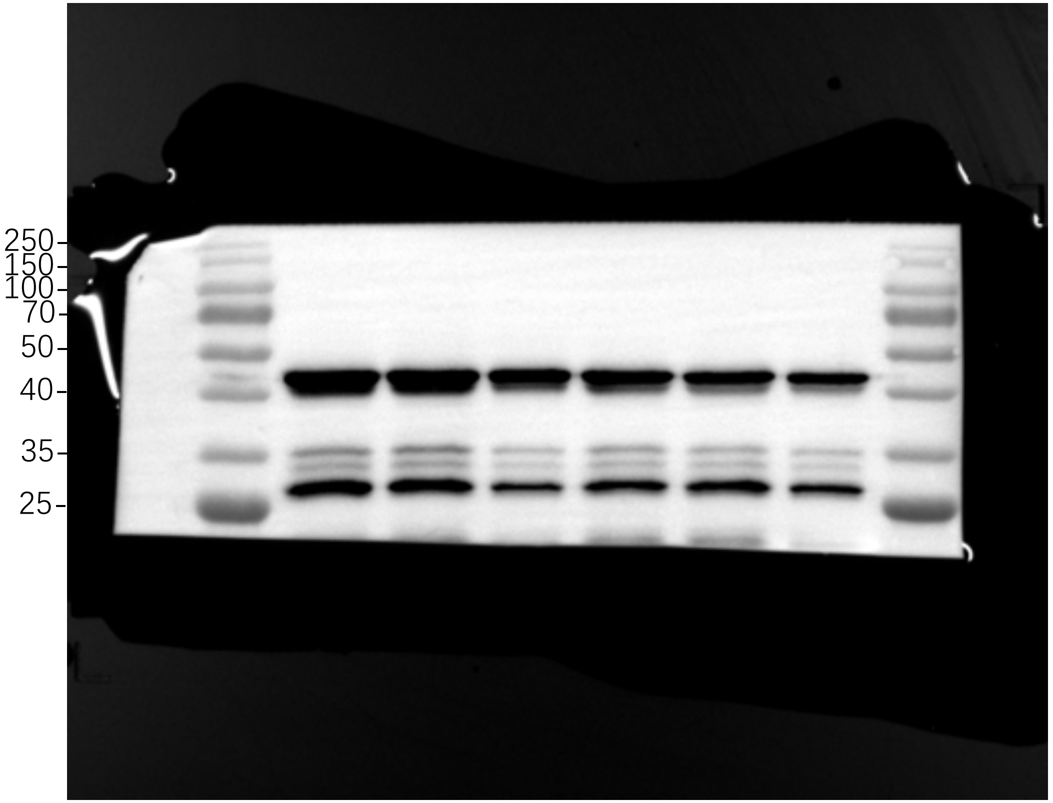

ErK1/2

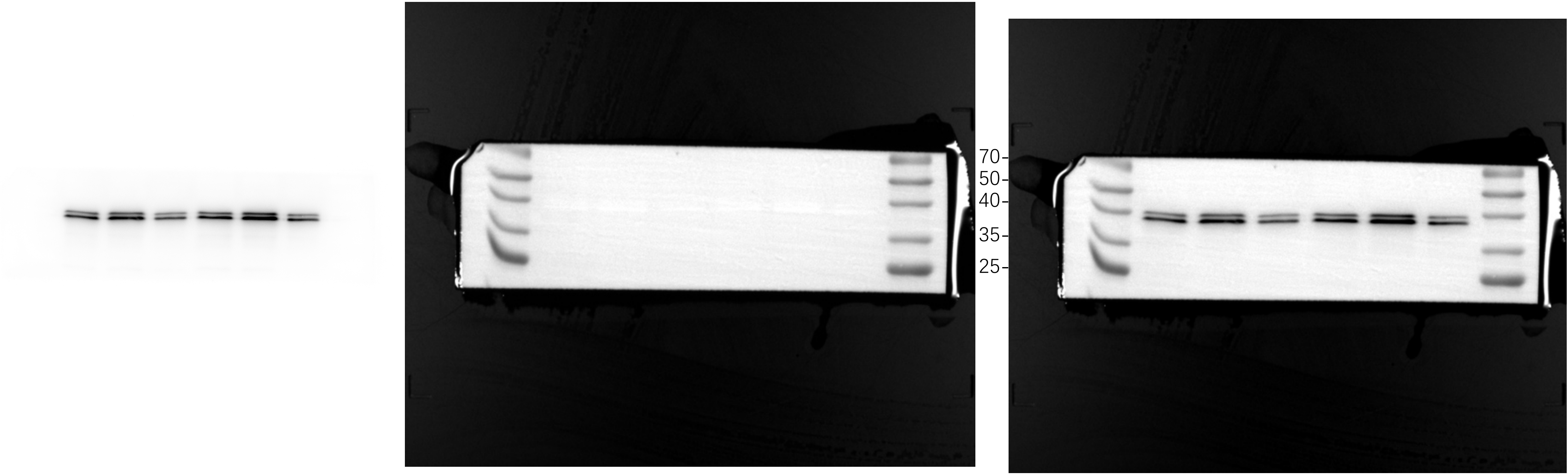

p-ErK1/2

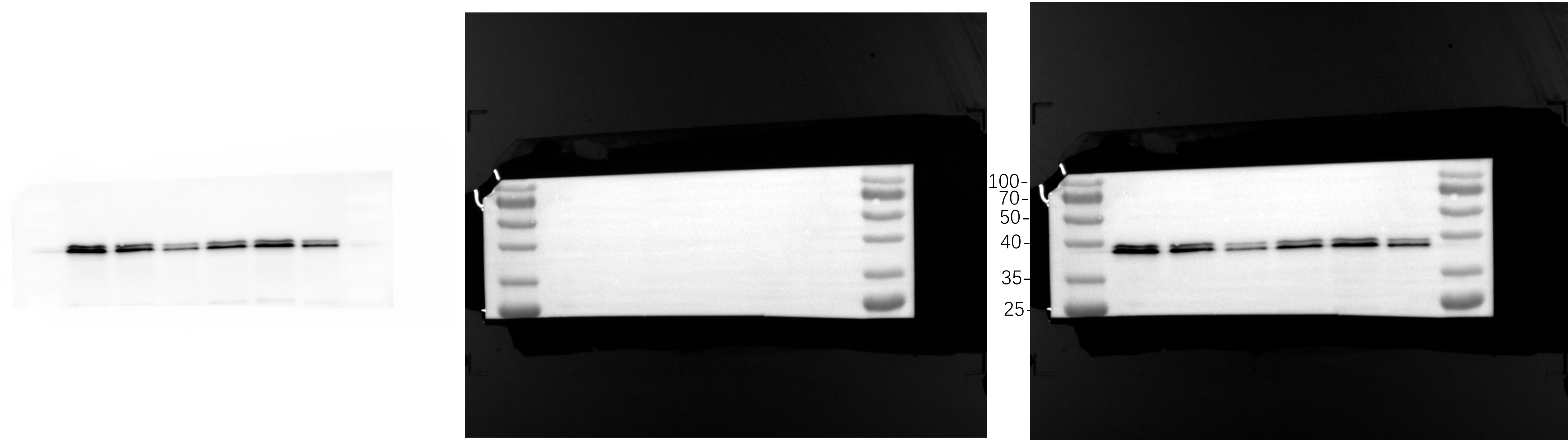

β-actin

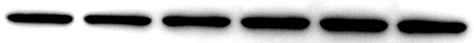

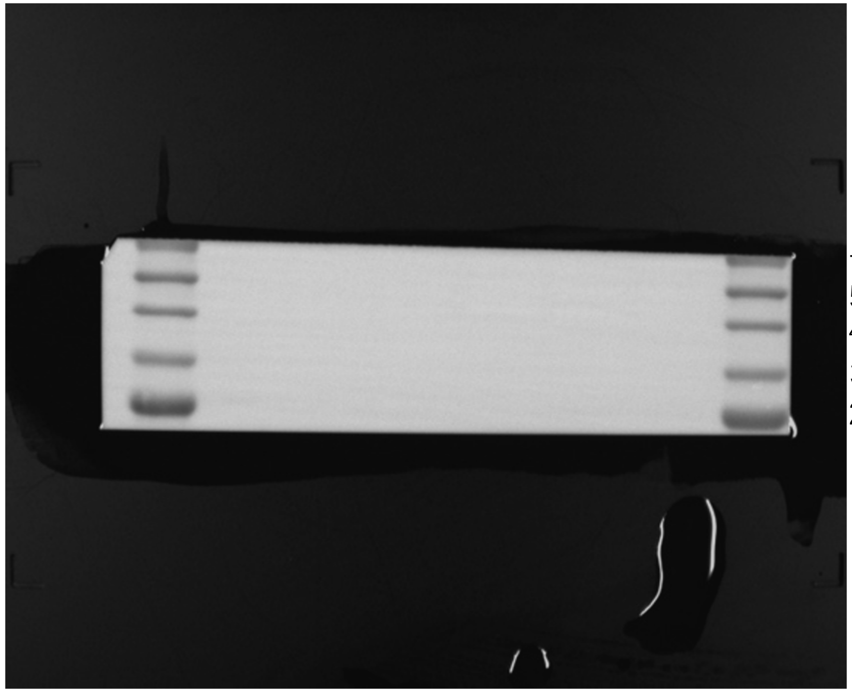

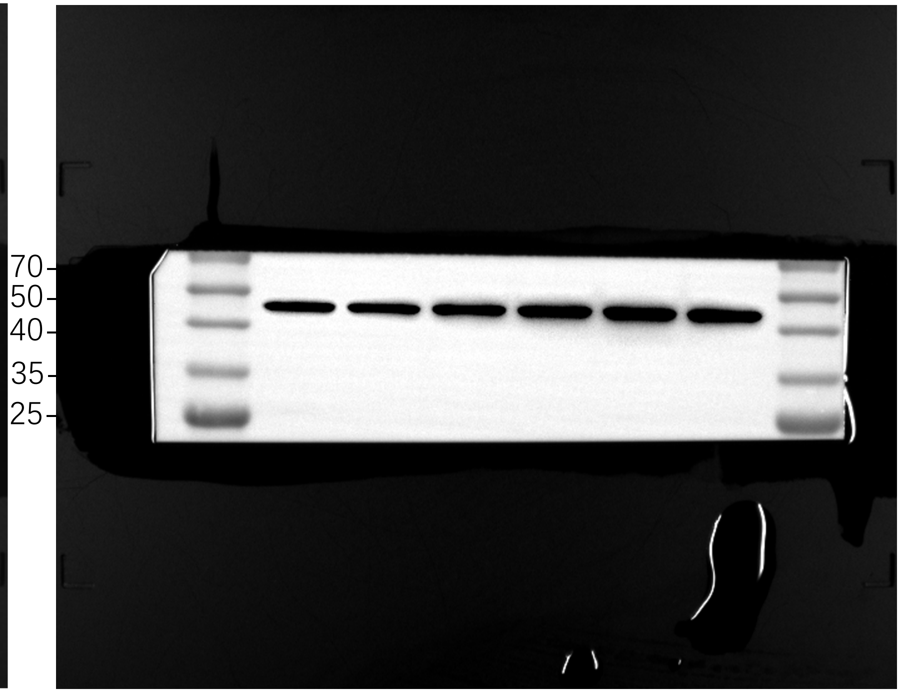

MEK1/2

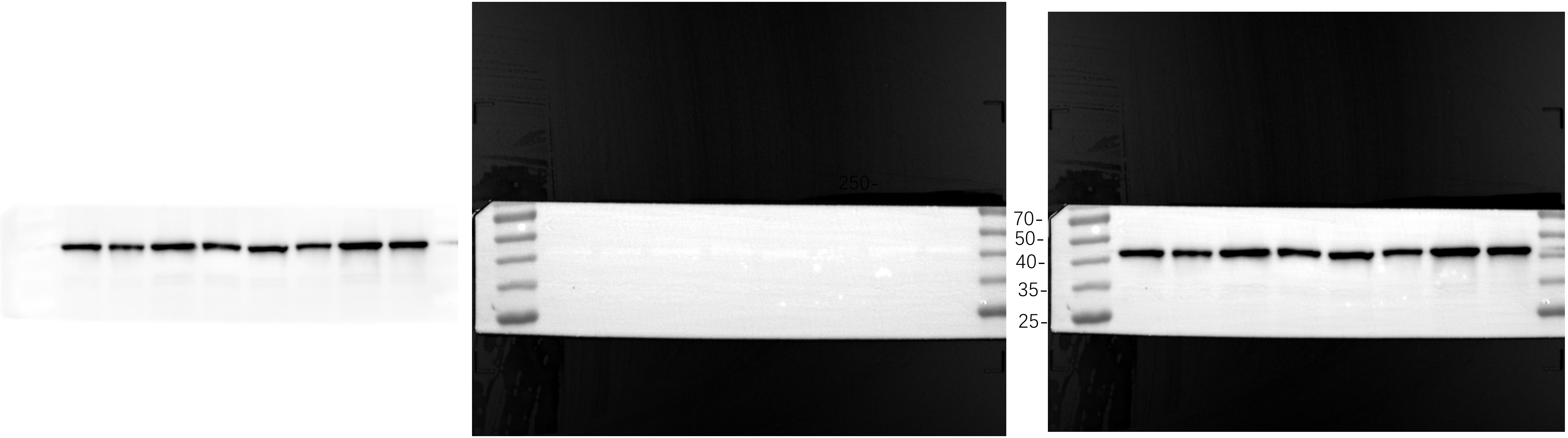

p-MEK1/2

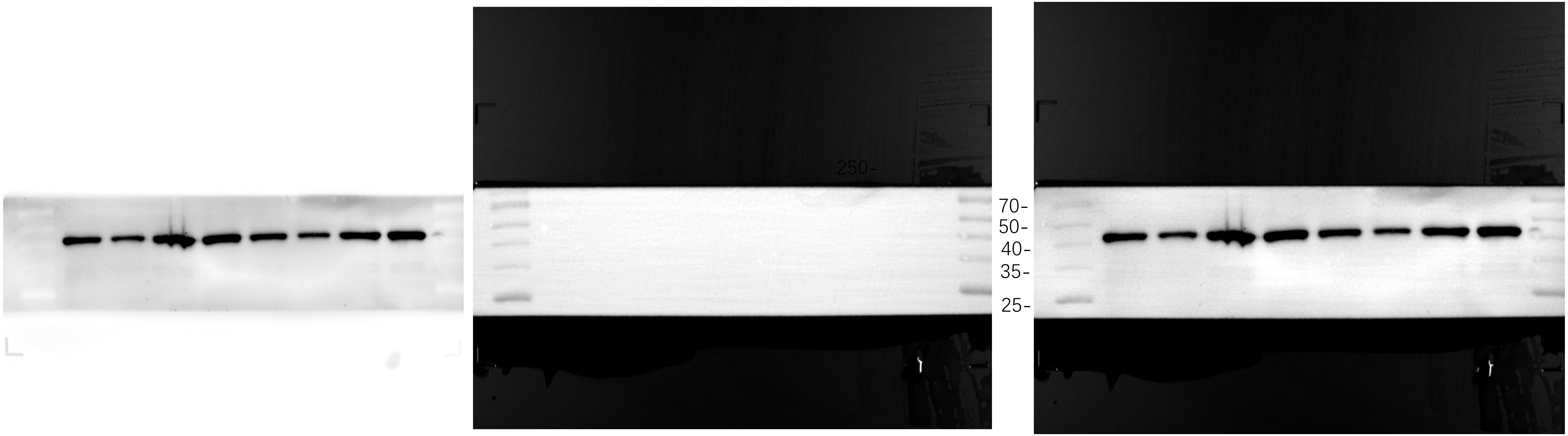

ErK1/2

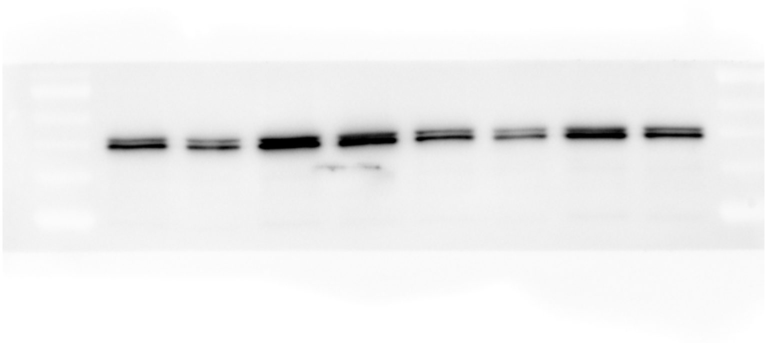

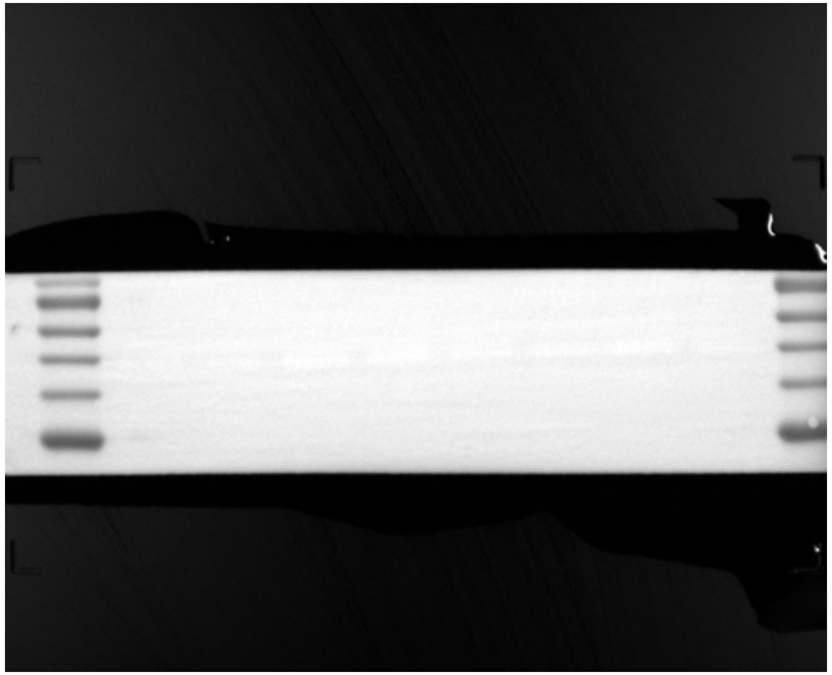

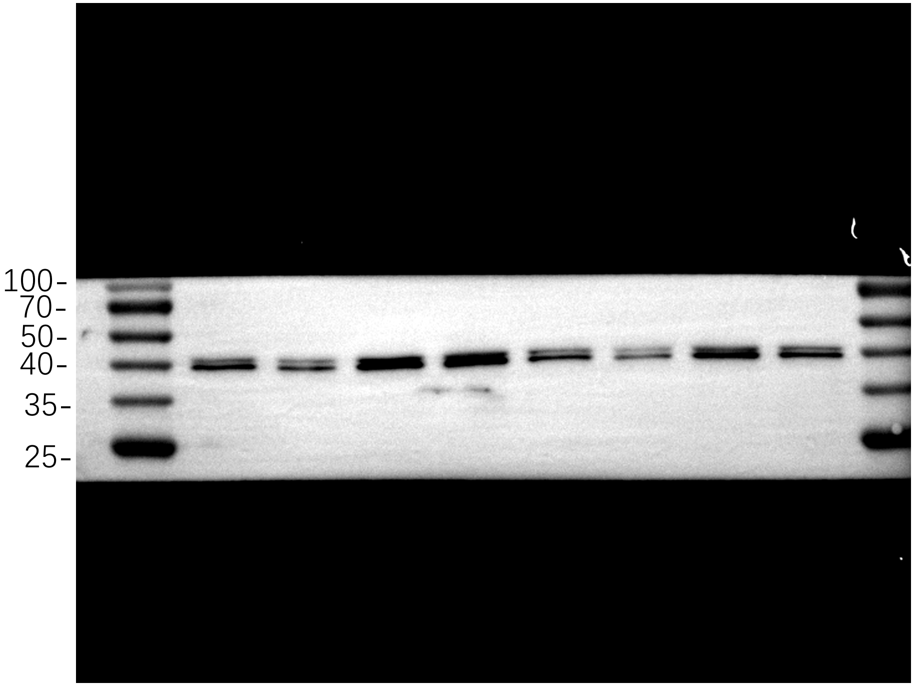

p-ErK1/2

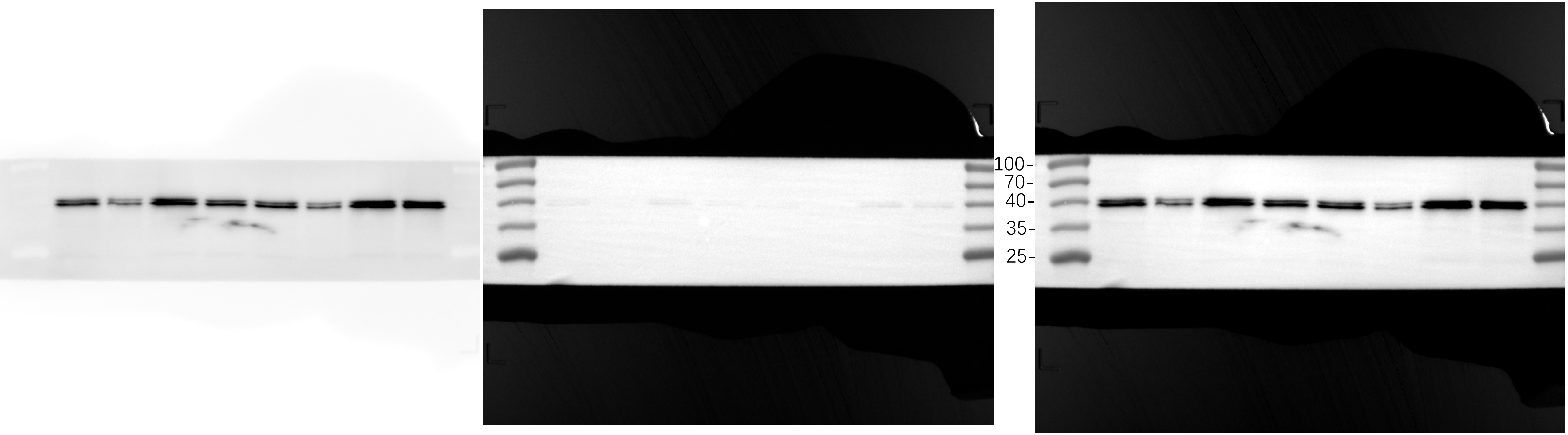

β-actin

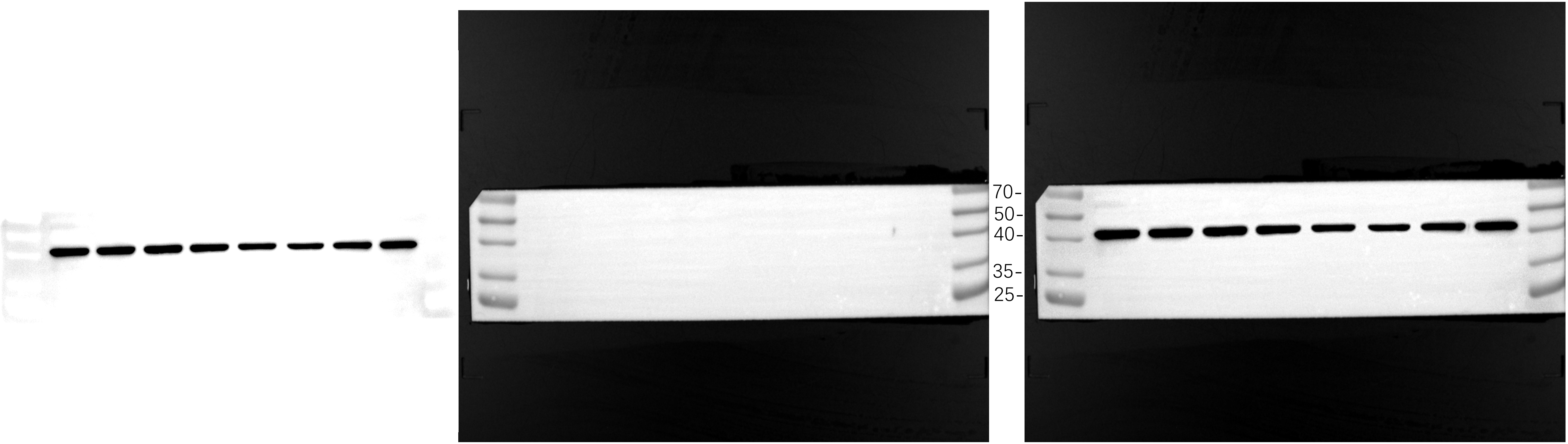

Supplement: Supplementary file 1 — Supplementary Files [file 41419_2022_4692_MOESM1_ESM.pdf]
